# Evolution and Expression Patterns of *TCP* Genes in Asparagales

**DOI:** 10.3389/fpls.2017.00009

**Published:** 2017-01-17

**Authors:** Yesenia Madrigal, Juan F. Alzate, Natalia Pabón-Mora

**Affiliations:** ^1^Facultad de Ciencias Exactas y Naturales, Instituto de Biología, Universidad de AntioquiaMedellín, Colombia; ^2^Centro Nacional de Secuenciación Genómica, Sede de Investigación Universitaria, Facultad de Medicina, Universidad de AntioquiaMedellín, Colombia

**Keywords:** *Cattleya trianae*, *CINCINNATA*, *CYCLOIDEA*, Hypoxidaceae, *Hypoxis decumbens*, floral symmetry, Orchidaceae, *PROLIFERATION CELL FACTOR*

## Abstract

*CYCLOIDEA-like* genes are involved in the symmetry gene network, limiting cell proliferation in the dorsal regions of bilateral flowers in core eudicots. *CYC-like* and closely related *TCP* genes (acronym for *TEOSINTE BRANCHED1, CYCLOIDEA*, and *PROLIFERATION CELL FACTOR*) have been poorly studied in Asparagales, the largest order of monocots that includes both bilateral flowers in Orchidaceae (ca. 25.000 spp) and radially symmetrical flowers in Hypoxidaceae (ca. 200 spp). With the aim of assessing *TCP* gene evolution in the Asparagales, we isolated *TCP-like* genes from publicly available databases and our own transcriptomes of *Cattleya trianae* (Orchidaceae) and *Hypoxis decumbens* (Hypoxidaceae). Our matrix contains 452 sequences representing the three major clades of *TCP* genes. Besides the previously identified *CYC* specific core eudicot duplications, our ML phylogenetic analyses recovered an early *CIN-like* duplication predating all angiosperms, two *CIN-like* Asparagales-specific duplications and a duplication prior to the diversification of Orchidoideae and Epidendroideae. In addition, we provide evidence of at least three duplications of *PCF-like* genes in Asparagales. While *CIN-like* and *PCF-like* genes have multiplied in Asparagales, likely enhancing the genetic network for cell proliferation, *CYC-like* genes remain as single, shorter copies with low expression. Homogeneous expression of *CYC-like* genes in the labellum as well as the lateral petals suggests little contribution to the bilateral perianth in *C. trianae*. *CIN-like* and *PCF-like* gene expression suggests conserved roles in cell proliferation in leaves, sepals and petals, carpels, ovules and fruits in Asparagales by comparison with previously reported functions in core eudicots and monocots. This is the first large scale analysis of *TCP-like* genes in Asparagales that will serve as a platform for in-depth functional studies in emerging model monocots.

## Introduction

As currently circumscribed the order Asparagales is a species-rich group comprising ca. 50% of all monocots, corresponding to 10–15% of flowering plants (Chase et al., 2009, 2016; Chen et al., 2013; Givnish et al., 2016). The most recent phylogenetic analyses in the monocots place Orchidaceae as sister to all other Asparagales (Chen et al., 2013). The family is divided into five subfamilies: Apostasioideae, Vanilloideae, Cypripedioideae, Orchidoideae, and Epidendroideae (Chase et al., 2015; Endress, 2016). The floral groundplan in Asparagales varies primarily in the floral symmetry and the number of stamens (Simpson, 2006). The floral morphology of Asparagales outside orchids consists of radially symmetrical, trimerous flowers with tepaloid perianth and free floral organs, although a few exceptions have been documented in *Aspidistra* (Asparagaceae), *Gethyllis* (Amaryllidaceae), *Neoastelia* (Asteliaceae) and *Pauridia* (Hypoxidaceae) (Rudall, 2002; Rudall and Bateman, 2002, 2004; Kocyan, 2007). Conversely, orchid flowers are variously bilateral and undergo extreme elaboration of some organs, including differentiation of perianth parts, stamen abortion, and fusion of floral parts from the same whorl or from different whorls (Rudall, 2002). In the bilateral resupinated orchid flowers the two dorsal petals are very similar to each other, whereas the ventral one (the lip or labellum) often undergoes extreme elaboration in shape, color, size and epidermal specializations (Rudall and Bateman, 2004; Pabón-Mora and González, 2008; Mondragón-Palomino and Theißen, 2009; Rudall et al., 2013; Endress, 2016). In the inner floral whorls bilateral symmetry is evident by the formation of a gynostemium that results from the congenital fusion between the single fertile stamen (sometimes two fertile stamens) and stigmas (Rudall and Bateman, 2002; Pabón-Mora and González, 2008; Endress, 2016). Such floral elaboration has been linked to extremely specialized pollination mechanisms and the exceedingly high diversification rates in Orchidaceae (Gong and Huang, 2009; Mondragón-Palomino and Theißen, 2009; Mondragón-Palomino, 2013).

The genetic network underlying bilateral floral symmetry has been assessed using *Antirrhinum majus* floral symmetry mutants (Luo et al., 1996). This network includes the differential dorsiventral expression of four transcription factors in the two-lipped flowers of this species. Three transcription factors, *CYCLOIDEA (CYC), DICHOTOMA (DICH)*, and *RADIALIS (RAD)* regulate cell division on the dorsal portion of the flower primordium and during dorsal petal and stamen primordia initiation. Additionally, *RAD* outcompetes *DIVARICATA* (*DIV*) for binding proteins in the dorsal side of the flower, restricting *DIV* function to the ventral and lateral petals (Almeida et al., 1997; Galego and Almeida, 2002; Raimundo et al., 2013). Thus, *cyc/dich* mutants show radially symmetrical ventralized flowers (Luo et al., 1996, 1999). Both, *CYC* and *DICH* genes belong to the *TCP* gene family (acronym for *TEOSINTE BRANCHED 1 -TB1*- from *Z. mays, CYCLOIDEA -CYC*- from *A. majus y PROLIFERATION CELL FACTOR 1* and *2* -*PCF1* and *PCF2*- from *Oryza sativa)* (Doebley et al., 1997; Kosugi and Ohashi, 1997; Luo et al., 1999). *RAD* and *DIV* belong to the *MYB* (*Myeloblastosis*) gene family (Luo et al., 1999; Galego and Almeida, 2002; Corley et al., 2005; Costa et al., 2005).

The *TCP* genes encode putative basic-Helix-Loop-Helix (bHLH) transcription factors (Cubas et al., 1999). The bHLH domain recognizes a consensus sequence GGNCCCAC/GTGGNCCC required for DNA binding and activation or repression of transcription (Kosugi and Ohashi, 2002; Martín-Trillo and Cubas, 2010). Gene evolution analyses have identified two large groups of *TCP* genes, namely Class I (which include *PCF* homologs) and Class II (containing the *CIN/CYC/TB1-like* genes) (Cubas et al., 1999; Damerval and Manuel, 2003; Reeves and Olmstead, 2003; Broholm, 2009; Mondragón-Palomino and Trontin, 2011). Additional large scale duplications (i.e., those occurring prior to the diversification of major inclusive hierarchical groupings) have been found within the *CYC* genes. Two rounds of duplication occurred specifically in core eudicots, resulting in *CYC1, CYC2*, and *CYC3* clades, and one duplication specific to monocots resulting in the *RETARDED PALEA 1 (REP1)* and *TEOSINTE BRANCHED 1 (TB1)* clades (Vieira et al., 1999; Damerval and Manuel, 2003; Howarth and Donoghue, 2006; Navaud et al., 2007; Yao et al., 2007; Mondragón-Palomino and Trontin, 2011). In addition, species specific duplications (i.e., those occurring in a single species) have also been reported, often linked to polyploidy (Ma et al., 2016). Non-core eudicot homologs are known as the *CYC-like* genes (Damerval et al., 2007; Preston and Hileman, 2012; Horn et al., 2015). Functional characterization has concentrated in *CYC2* orthologs in eudicots, including Asterales, Brassicales, Dipsacales, Fabales, Lamiales and Malpighiales, among others (Busch and Zachgo, 2007; Gao et al., 2008; Preston et al., 2009; Wang et al., 2010; Zhang et al., 2010, 2013; Howarth et al., 2011; Tähtiharju et al., 2012; Yang et al., 2012). These studies have found *CYC2* expression restricted to the same dorsal floral domain and a conserved role as cell proliferation repressors resulting in bilateral symmetry (reviewed in Hileman, 2014). Fewer studies have been made in basal eudicots, but dissymmetric Fumarioids (Papaveraceae) do have asymmetric expression of *CYC-like* genes, suggesting that *CYC-like* recruitment to form bilateral flowers has occurred independently several times in eudicots (Damerval et al., 2007, 2013).

Less is known about the role of pre-duplication *CYC-like* genes in monocots (Bartlett and Specht, 2011; Mondragón-Palomino and Trontin, 2011; Preston and Hileman, 2012). Expression analyses of *CYC-like* genes in *Costus* (Costaceae; Zingiberales) and *Commelina* (Commelinaceae; Commelinales) suggest that they play a role in bilateral symmetry (Bartlett and Specht, 2011; Preston and Hileman, 2012). Functional studies in *O. sativa* (Poaceae, Poales) confirm that these genes contribute to the asymmetric growth of the dorsal *versus* the ventral portions of the flower, as shown by the *rep1* mutants which exhibit a smaller palea due to cell division arrest (Yuan et al., 2009). The only two studies available in Orchidaceae are particularly intriguing as they show very different expression patterns of *CYC/TB1-like* orthologs. Whereas, the only copy of *CYC/TB1-like* in *Orchis italica (OitaTB1)* is expressed exclusively in leaves (De Paolo et al., 2015), two of three *CYC/TB1-like* copies in *Phalaenopsis equestris, PeCYC1* and *PeCYC2*, seem to be expressed in higher levels (2–10 times more) in the dorsal sepals and the labellum compared to the ventral sepal and the lateral petals (Lin et al., 2016). Furthermore, some authors have hypothesized that the expression gradient of *TCP* genes is largely controlled by upstream expression of the *AP3/DEF* petal-stamen identity genes, resulting in higher concentrations of *CYC/TB1-like* genes in the dorsal floral regions; however, more experimental data is needed to support this (Mondragón-Palomino and Theißen, 2009).

It is unclear whether closely related *TCP-like CINCINNATA* (*CIN*) and *PROLIFERATION CELL FACTOR* (*PCF*) genes play any role in floral symmetry. *CIN* was originally characterized in *A. majus* and more recently in *Arabidopsis thaliana* (Crawford et al., 2004; Nag et al., 2009; Sarvepalli and Nath, 2011; Danisman et al., 2013). In both species *CIN* controls cellular proliferation in petals and cellular arrest in leaves (Crawford et al., 2004; Nag et al., 2009). On the other hand, *O. sativa PCF1* and *PCF2* are involved in axillary meristem repression, likely *via* the activation of *PROLIFERATING CELL NUCLEAR ANTIGEN (PCNA)*, which encodes a protein involved in DNA replication and repair, maintenance of chromatin structure, chromosome segregation, and cell-cycle progression (Kosugi and Ohashi, 1997). Other studies in *A. thaliana* suggest that *PCF-like* genes are also involved in gametophyte development, transduction of hormonal signals, mitochondrial biogenesis, leaf and flower morphogenesis, seed germination, branching, and even circadian clock regulation (Koyama et al., 2007; Pruneda-Paz et al., 2009; Giraud et al., 2010; Kieffer et al., 2011; Resentini et al., 2015). Recent studies in the model orchid *P. equestris* have found that *PeCIN8* (*CIN-like*) and *PePCF10* (*PCF-like*) control cell proliferation and cell shape in petals, ovules and leaves (Lin et al., 2016).

In order to study the contribution of *CYC/TB1-like* and the closely related *CIN-like* and *PCF-like* genes to floral patterning in Asparagales, we first determined copy number and characteristic protein motifs and then assessed gene lineage evolution including a vast sampling of *TCP-like* genes across angiosperms and particularly of Asparagales monocots. Next, we evaluated the expression patterns of all *TCP-like* genes in dissected floral organs, young leaves, and fruits of *Hypoxis decumbens* (Hypoxidaceae) which has typical asparagalean radial trimerous flowers with free parts, and *Cattleya trianae* (Orchidaceae), that has bilateral flowers, and a single fertile stamen fused with the tree stigmas (i.e., gynostemium). Finally, we propose hypotheses on functional evolution based on previous literature reports and comparisons with our results that suggest different trends among gene clades when comparing Asparagales to model core eudicots.

## Materials and methods

### Gene isolation and phylogenetic analyses

In order to isolate putative *TCP-like* homologs in Asparagales, searches were performed using previously reported *TCP* genes from eudicots, monocots and in particular Orchidaceae as queries (Mondragón-Palomino and Trontin, 2011; Preston and Hileman, 2012; De Paolo et al., 2015; Horn et al., 2015). Searches included homologs from all the three main clades of *TCP* genes: *CYC-like, CIN-like* and *PCF-like*. Searches were done using BLAST tools (Altschul et al., 1990) in the orchid specific available databases including Orchidbase 2.0 (http://orchidbase.itps.ncku.edu.tw/) (Tsai et al., 2013), Orchidstra (http://orchidstra2.abrc.sinica.edu.tw/orchidstra2/index.php) (Su et al., 2013), as well as the more inclusive OneKP database (http://www.bioinfodata.org/Blast4OneKP/). All core eudicot sequences, were isolated from Phytozome (https://phytozome.jgi.doe.gov/pz/portal.html) and genbank (https://www.ncbi.nlm.nih.gov/genbank/).

In addition to the available databases we generated two transcriptomes from *C. trianae* (Orchidaceae) and *H. decumbens* (Hypoxidaceae). The transcriptome for each species was generated from mixed material from 3 biological replicates and included vegetative and reproductive meristems, floral buds, young leaves and fruits in as many developmental stages as possible. Total RNA was purified and used for the preparation of one mRNA (polyA) HiSeq library for each species. RNA-seq experiments were conducted using truseq mRNA library construction kit (Illumina, San Diego, California, USA) and sequenced in a HiSeq 2000 instrument reading 100 base paired end reads.

The transcriptome was assembled *de novo* with Trinity v2 following default settings. Read cleaning was performed with prinseq-lite v0.20.4 with a quality threshold of Q35 and a minimum read length of 50 bases. Contig metrics are as follows: (1) *H. decumbens* total assembled bases: 73,787,751; total number of contigs (>101 bases): 157,153; average contig length: 469 bp; largest contig: 15,554 bp; contig N50: 1075 bp; contig GC%: 46,42. (2) *C. trianae* total assembled bases: 63,287,862 bp; total number of contigs (>101 bases): 109,708; average contig length: 576 bp; largest contig: 9321 bp; contig N50: 1401 bp; contig GC%: 42,73. Homologous gene search was performed using BLASTN with the query sequences downloaded from GenBank and other databases (see above). In order to estimate the relative abundance of the assembled contigs, cleaned reads were mapped against the *de novo* assembled dataset and counted with two different strategies. The first one involved the mapping algorithm of the software Newbler v2.9 where only the unique matching read pairs were accepted as positive counts. The second one involved the mapping algorithm BOWTIE2 and raw reads counts as well as RPKM were calculated to each assembled contig (Table 1).

**Table 1:** **Selected features of ***TCP*** and ***AP3-like*** gene contigs taken from sequences and the transcriptomes of ***Cattleya trianae*** and ***Hypoxis decumbens*****.

**Gene CDS**	**Contig**	**CDS size (in bp)**	**Protein size (in AA)**	URP^*^ Newbler	RRC^**^ RPKM	**RPKM**	**RPKM rank**
CtrAP3a	c19830_g1_i1	684	227	4047	3877	91,02338579	1689
CtrAP3b	c16430_g1_i2	696	231	15	719	16,64198541	7551
CtrAP3c	c16430_g1_i1	669	222	184	1002	23,72478436	5775
CtrAP3d	c14728_g2_i1	628	208	1218	1228	38,72393988	3786
CtrAP3e	c13983_g1_i1	601	199	321	269	9,445413314	11262
CtrCIN1	c10815_g1_i1	945	314	565	540	7,473218106	13078
CtrCIN2	c20571_g1_i1	975	324	5413	5455	58,71380149	2597
CtrCIN3	c20515_g2_i1	1158	385	2065	2091	28,27740553	4987
CtrCIN4	c20515_g1_i1	1116	371	1436	880	13,36057701	8882
CtrCIN5	c19791_g1_i1	1116	371	530	491	6,141835864	14794
CtrCIN6	c18629_g1_i1	957	319	453	411	8,008505294	12506
CtrPCF1	c14669_g1_i1	930	309	260	258	6,560658931	14228
CtrPCF2	c10284_g1_i1	759	252	306	308	8,318287473	12198
CtrPCF3	c22906_g1_i1	882	293	1109	1118	23,04243274	5910
CtrPCF4	c10751_g2_i1	561	187	315	230	11,37525956	9984
CtrPCF5	c21320_g2_i1	900	299	493	401	6,065023082	14927
CtrPCF6	c23464_g2_i1	828	275	5821	928	15,39001893	7984
CtrPCF7	c13613_g1_i1	684	227	1023	1050	25,7820899	5397
CtrPCF8	c8381_g1_i1	642	213	136	136	3,198372774	21993
CtrPCF9	c16850_g1_i1	1032	343	1021	529	6,68019211	14059
CtrPCF10	c24243_g4_i1	1107	368	269	140	2,935430718	23098
CtrPCF11	c20858_g1_i1	963	320	181	171	3,452818247	21033
CtrTB1	c62044_g1_i1/c74262_g1_i1	330	110	6/15	7/14	0,507584894922/0,584445576	58492/52988
HydDEF1a	c29923_g1_i2	181	59	175	8	1,074179469	58639
HydDEF1b	c29923_g1_i4	684	227	80	84	3,992750518	25641
HydDEF2	c23342_g1_i1	672	223	533	513	19,98971549	6376
HydCIN1	c26829_g2_i1	717	239	605	420	12,7762255	9853
HydCIN2	c25446_g2_i3	1272	424	496	589	17,24524272	7370
HydCIN3	c25446_g2_i4	651	217	2655	338	17,37878284	7322
HydCIN4	c25446_g2_i2	858	286	1355	1314	44,60569165	2753
HydPCF1	c25819_g1_i2	1038	345	12	266	7,587936907	15736
HydPCF2	c25819_g1_i1	939	312	11	236	8,050262478	14962
HydPCF3	c29001_g5_i3	300	100	75	64	5,51807531	20195
HydPCF4	c29001_g5_i1	387	129	23	20	1,853254689	41814
HydPCF5	c29001_g5_i4	408	136	32	43	3,099053674	30449
HydPCF6	c29001_g5_i2	411	137	42	57	4,208770608	24720
HydPCF7	c27872_g4_i1	771	256	732	896	26,84914255	4743
HydPCF8	c27872_g3_i2	762	253	334	528	16,30864126	7759
HydPCF9	c27872_g3_i1	756	251	108	181	6,950127044	16867
HydPCF10	c27872_g2_i1	675	225	138	157	9,763071436	12576
HydTB1	c52607_g1_i1	258	85	3	3	0,364509027	119496

* *Unique read pairs - number of specific/unique read pairs supporting each contig*.

** *Raw read counts*.

All sequences isolated were compiled with Bioedit (http://www.mbio.ncsu.edu/bioedit/bioedit.html). Sequences shorter than 200 bp lacking similarity with a region of the putative bHLH motif were discarded. Nucleotide sequences were subsequently aligned using the online version of MAFFT (http://mafft.cbrc.jp/alignment/software/) (Katoh et al., 2002) with a gap open penalty of 3.0, offset value of 1.0 and all other default settings. The alignment was then refined by hand using Bioedit considering as a reference the 60–70 aa reported as conserved in the TCP protein domain (Cubas et al., 1999). To better understand the evolution of the *TCP* gene lineage, and to integrate previous eudicot and monocot specific phylogenetic analyses (Damerval and Manuel, 2003; Hileman and Baum, 2003; Reeves and Olmstead, 2003; Howarth and Donoghue, 2006; Damerval et al., 2007; Bartlett and Specht, 2011; Mondragón-Palomino and Trontin, 2011; Preston and Hileman, 2012; Horn et al., 2015), we performed Maximum likelihood (ML) phylogenetic analyses using the nucleotide sequences with RaxML-HPC2 BlackBox through the CIPRES Science Gateway (https://www.phylo.org/) (Miller et al., 2010). Bootstrapping (BS) was performed according to the default criteria in RAxML (200–600 replicates). The *PCF-like* gene from *Amborella trichopoda (AtrTCP4)* as well as all other *PCF-like* sequences were used as the outgroup. To find the molecular evolution model that best fit our data, we used the jModelTest package implemented in MEGA6 (Posada and Crandall, 1998). Trees were observed and edited using FigTree v1.4.0 (http://tree.bio.ed.ac.uk/software/figtree/) (Rambaut, 2014). Newly isolated sequences from our own generated transcriptomes from *C. trianae* (Orchidaceae) and *H. decumbens* (Hypoxidaceae) can be found under Genbank numbers KY296315–KY296347. All sequences included in the phylogenetic analyses can be found in the Supplementary Table 1.

### Identification of new protein motifs

In order to detect previously reported, as well as to identify new, conserved motifs, 77 *TCP-like* genes were selected representing major model eudicot and monocot groups of this study (i.e., *A. majus, O. sativa, Aloe vera, P. equestris, C. trianae* and *H. decumbens*). Sequences were permanently translated and uploaded as amino acids to the online MEME server (http://meme-suite.org/tools/meme) and run with all the default options (Bailey et al., 2006). Specific analyses for each of the *TCP* clades (i.e., *CYC-like, CIN-like, PCF-like*) were also performed.

### Expression analyses by RT-PCR

To examine and compare the expression patterns of *TCP-like* genes we used floral buds, dissected floral organs, leaves, and fruits of *C. trianae* and *H. decumbens*. Preanthetic floral buds of *H. decumbens* were dissected into sepals, petals, stamens and carpels. In addition whole floral buds, inmature fruits (F1, right after tepals shed off), mature fruits (F2, before lignification), and young leaves were also collected. Preanthetic floral buds of *C. trianae* were dissected into sepals, lateral petals, labellum (or lip), gynostemium, and ovary. Young leaves were also collected. Total RNA was isolated from each organ collected using the SV Total RNA Isolation System kit (Promega, Madison, WI, USA), and resuspended in 20 μl of DEPC water. RNA was treated with DNAseI (Roche, Basel, Switzerland) and quantified with a NanoDrop 2000 (Thermo Scientific, Waltham, MA) (Wilfinger et al., 1997). Three Micrograms of RNA were used as a template for cDNA synthesis (SuperScriptIII RT, Invitrogen) using OligodT primers. The cDNA was diluted 1:4 for amplification reactions by RT-PCR. Primers were designed in specific regions like portions flanking the conserved domains for each copy found in *C. trianae* and *H. decumbens* (Supplementary Table 2). Each amplification reaction incorporated 9 μl of EconoTaq (Lucigen, Middleton, WI), 6 μl of nuclease free water, 1 μl of BSA (5 μg/ml), 1 μl of Q solution (5 μg/μl) 1 μl fwd primer (10 mM), 1 μl rev primer (10 mM), and 1 μl of template cDNA for a total of 20 μl. Thermal cycling profiles followed an initial denaturation step (94°C for 30 s), an annealing step (50–59°C for 30 s) and an extension step with polymerase (72°C for 10 min), all by 30–38 amplification cycles. *ACTIN2* was used as a load control. PCR products were run on a 1.0% agarose gel stained with ethidium bromide and digitally photographed using a Whatman Biometra® BioDoc Analyzer.

## Results

Exhaustive search from available databases retrieved 452 *TCP* Class I and Class II sequences from flowering plants. From these 138 belong to the Orchidaceae, including 18 homologs from *C. trianae*; and 110 sequences belong to non-Orchidaceae Asparagales, including 15 homologs from *H. decumbens* (Supplementary Table 1).

**Figure 1:**
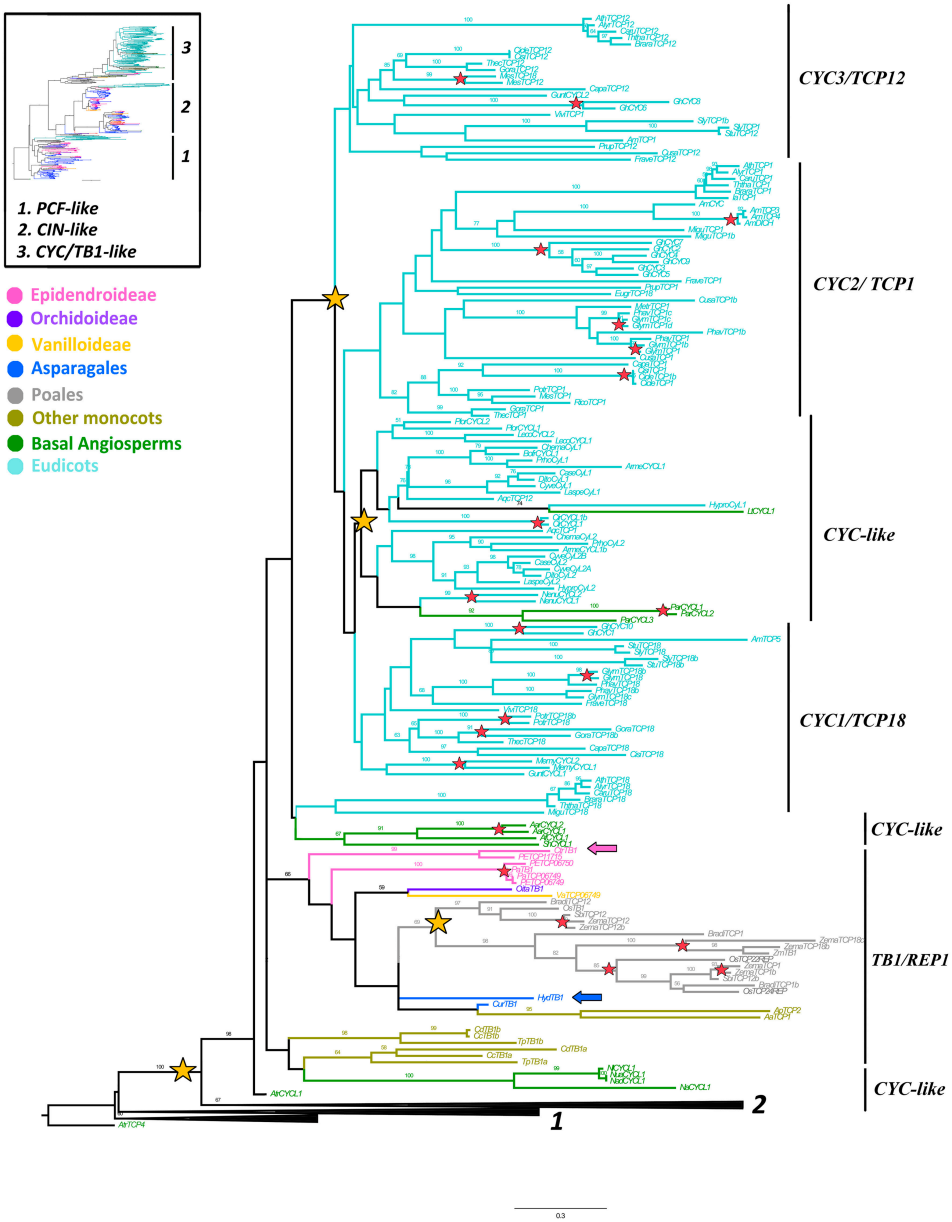
**ML analysis of ***CYC/TB1-like*** genes**. Overview (upper left) summary Maximum Likelihood (ML) analysis of all *TCP-like* genes, where (1) corresponds to *PCF-like* homologs, (2) to the *CIN-like* clade and (3) to the *CYC/TB1-like* clade. Detailed (right) gene tree expanded in the *CYC/TB1-like* clade (3); yellow stars indicate large-scale duplication events in core, basal eudicots and monocots; resulting in the core eudicot clades *CYC3/TCP12, CYC2/TCP1* and *CYC1/TCP18*, the basal eudicot *CYC-like* clades and the *TB1* and *REP1* clades in Poales; red stars indicate species-specific duplication events; blue and pink arrows indicate *H. decumbens* and *C. trianae* homologs, respectively; branch and taxa colors correspond to those in the overview tree to the upper left. BS values ≥ 50 are shown.

ML analyses were performed using the complete nucleotide sequences of all *TCP-like* genes isolated. The *A. trichopoda AtrTCP4* together with all other isolated *PCF-like* genes were used as the outgroup. The analysis recovered two clades previously reported in *TCP* genes, namely the *CYC/TB1-like* clade (with a Bootstrap Support, BS = 98) and the *CIN-like* clade (BS = 67) (Figure 1). We will discuss our results for each clade separately.

### *CYC/TB1-like* gene evolution

We were able to isolate 168 sequences belonging to the *CYC/TB1-like* clade (Supplementary Table 1). Our sampling includes 15 sequences from four species of Poales, 10 sequences from seven species of Asparagales (incl. Orchidaceae), eight from five species of Commelinales (*Commelina, Alstroemeria, Tradescantia*), 14 from nine species of basal angiosperms and 121 from 46 species of eudicots. Only one homolog from *Curculigo* spp. (*CurTB1*) and one homolog from *H. decumbens* (*HydTB1*) were recovered from our blast searches. Moreover, an exhaustive search in Orchidaceae specific databases resulted in eight additional copies: three from *P. equestris* (*PETCP06750, PETCP06749, PETCP11715*), two from *P. aphrodite* (*PaTB1, PaTCP06749*) and one from *C. trianae* (*CtrTB1*) (Epidendroideae); one homolog from *O. italica* (*OitaTB1*) (Orchidoideae); and one homolog from *Vanilla shenzhenica* (*VaTCP06749*) (Vanilloideae). *CYC/TB1-like* homologs seem to have undergone size reduction in Asparagales, and only the searches made with monocot *TB1* genes yielded positive hits. Interestingly, in the two transcriptomes newly obtained in the present research, the *CYC*/*TB1-like* contigs were supported by fewer reads (when compared to *MADS-box APETALA3* floral organ identity genes and other *TCP-like* genes; Table 1) suggesting low expression of these transcripts.

The resulting ML topology recovered the three previously established core eudicot subclades (Howarth and Donoghue, 2006) with very low support (BS < 50), namely *CYC1/TCP18, CYC2/TCP1*, and *CYC3/TCP12* (Figure 1). Our analysis also recovers the previously identified duplication of *CYC-like* genes in basal eudicots (Citerne et al., 2013), and another in Poales, the latter resulting in the *REP1/TB1* clades (Mondragón-Palomino and Trontin, 2011). Most basal angiosperms and many monocots outside Poales have single copy *CYC-like* genes that predate the independent duplications in eudicots and Poales (Figure 1). Intraspecific duplications in monocots have occurred in *Zea mays*, as well as in the orchids *P. equestris* and *P. aphrodite* (Figure 1; BS = 100). Outside of the monocots, local specific duplications have also occurred in the basal angiosperms *Aristolochia ringens* and *Persea americana*, in the basal eudicots *Circaeaster* and *Nelumbo*, and in core eudicots such as *Gerbera, Antirrhinum, Citrus, Glycine, Populus*, and *Gossypium* (Figure 1).

**Figure 2:**
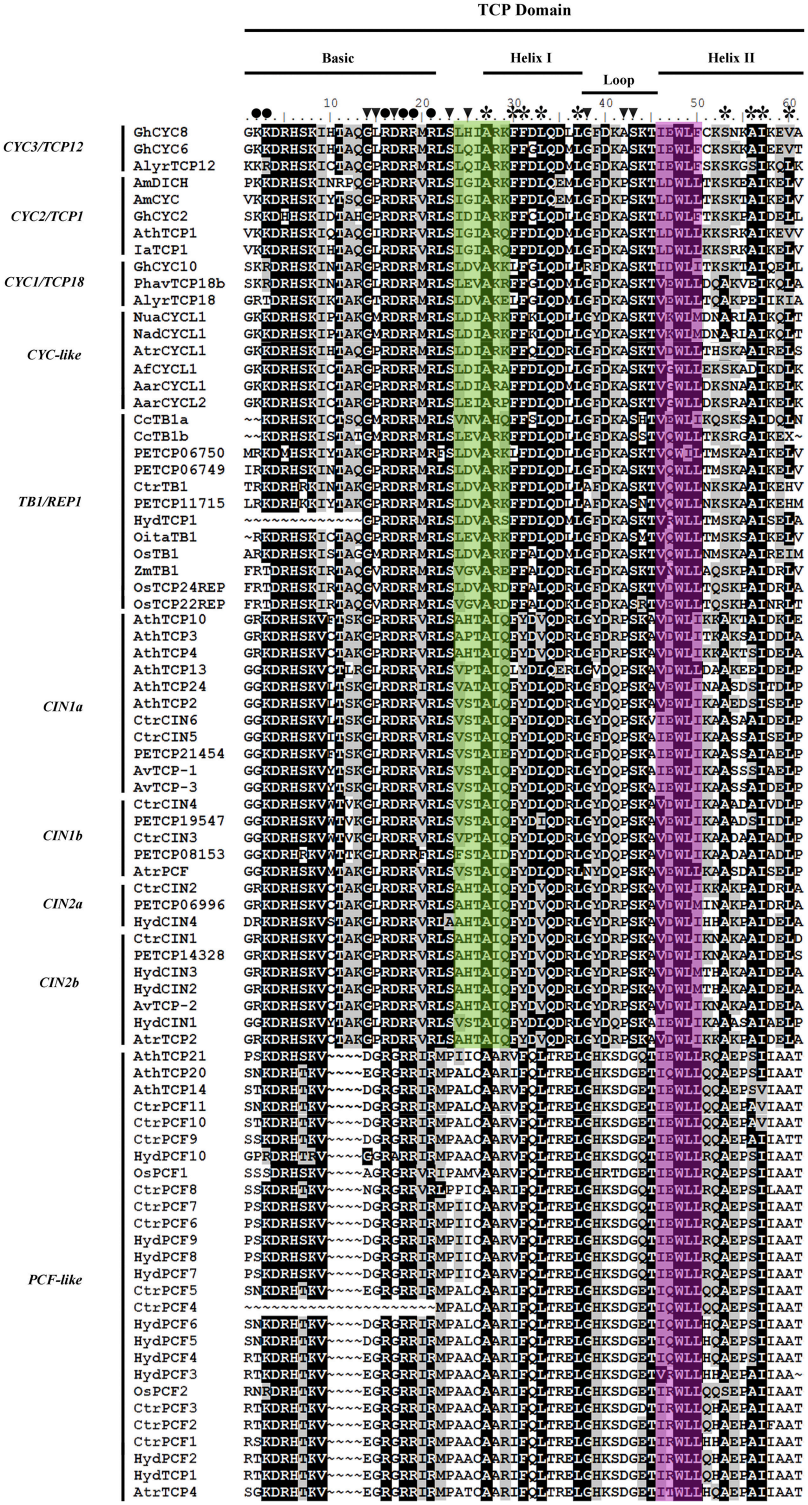
**TCP protein domain alignment with Asparagales representative sequences**. *Oryza sativa* (Poales) was used for reference. Names to the left indicate the clade to which sequences belong according to Figures 1, 4, 5 and Supplementary Table 1. The upper bars point to the putative structure bHLH (basic-Helix-Loop-Helix) at the TCP domain. Circles indicate residues forming part of the putative bipartite NLS; asterisks indicate conserved hydrophobic residues in the helices; black arrowheads point to residues (glycine or proline) that disrupt α-helix formation (modified following Cubas et al., 1999). The green box indicates changes in residues between CYC-like and CIN-like proteins. The pink box indicates the LxxLL motif with significant variations outside the CYC2 clade.

Members of the *CYC/TB1-like* clade show very little variation in the ca. 60 amino acid TCP domain (*sensu* Cubas et al., 1999) consisting of a putative basic-Helix-Loop-Helix (bHLH) domain (Figure 2). We were able to identify the highly conserved residues previously reported for the putative bipartite Nuclear Localization Signal (NLS) at the N-terminus flanking the bHLH, which provide hydrophobicity in the α-helices and in the loop region between the two. The loop itself is highly conserved in all CYC/TB1-like sequences except for AmDICH and AmCYC that have an A > P change at position 42. The second helix contains the LxxLL motif in all CYC2 proteins; this motif is modified into a VxWLx motif in other CYC-like proteins.

Our MEME analysis identified motifs 1 and 2 corresponding to the TCP domain (Supplementary Figure 1). At the start of Helix I, between positions 24–29 we found specific amino acids exclusive to CYC protein homologs. Outside the TCP domain, motifs 7 and 10 (reported also by Bartlett and Specht, 2011) and motifs 36–40, 42, and 43 (reported also by De Paolo et al., 2015) were recovered in our analysis as conserved in all CYC proteins. In addition, the protein interaction R domain (motif 11) putatively involved in hydrophilic α-helix formation in TCP Class II genes (shared between CYC and CIN proteins) was also identified (Supplementary Figure 1; Cubas et al., 1999). However, this motif is absent from CtrTB1 and REP-1 (Supplementary Figure 1; Yuan et al., 2009). Previously unidentified motifs include motif 4, exclusive to Epidendroideae and motif 5, exclusive to *Phalaenopsis* species. Whereas, most orchid CYC-like proteins (including *O. italica* OitaTB1 and *C. trianae* CtrTB1) do not share any common motifs with the canonical *A. majus* paralogs outside the TCP domain, the *Phalaenopsis* CYC-like homolog (PETCP11715) shares motifs 6, 9, and 12 with AmDICH or AmCYC.

### *CIN-like* gene evolution

A total of 155 *CIN-like* homologs were recovered and unlike the *CYC-like* sampling, most *CIN-like* sequences belong to the Asparagales (Supplementary Table 1). Our sampling contains 57 sequences from 35 non-Orchidaceae Asparagales species, including four paralogs from *H. decumbens* labeled *HydCIN1-HydCIN4*. A total of 78 *CIN-like* homologs were isolated, including four homologs from two Apostasioideae species, nine homologs from three Vanilloideae species, five homologs from three Cypripedioideae species, 22 homologs from seven Orchidoideae species and 38 homologs from 11 Epidendroideae species. Furthermore, six paralogs were identified in *C. trianae* labeled *CtrCIN1-CtrCIN6*. Searches outside Asparagales were restricted to six homologs from *O. sativa*, two from *A. trichopoda* and 12 homologs from five eudicots species, including the canonical *A. majus CINCINNATA* (*AmCIN*).

**Figure 3:**
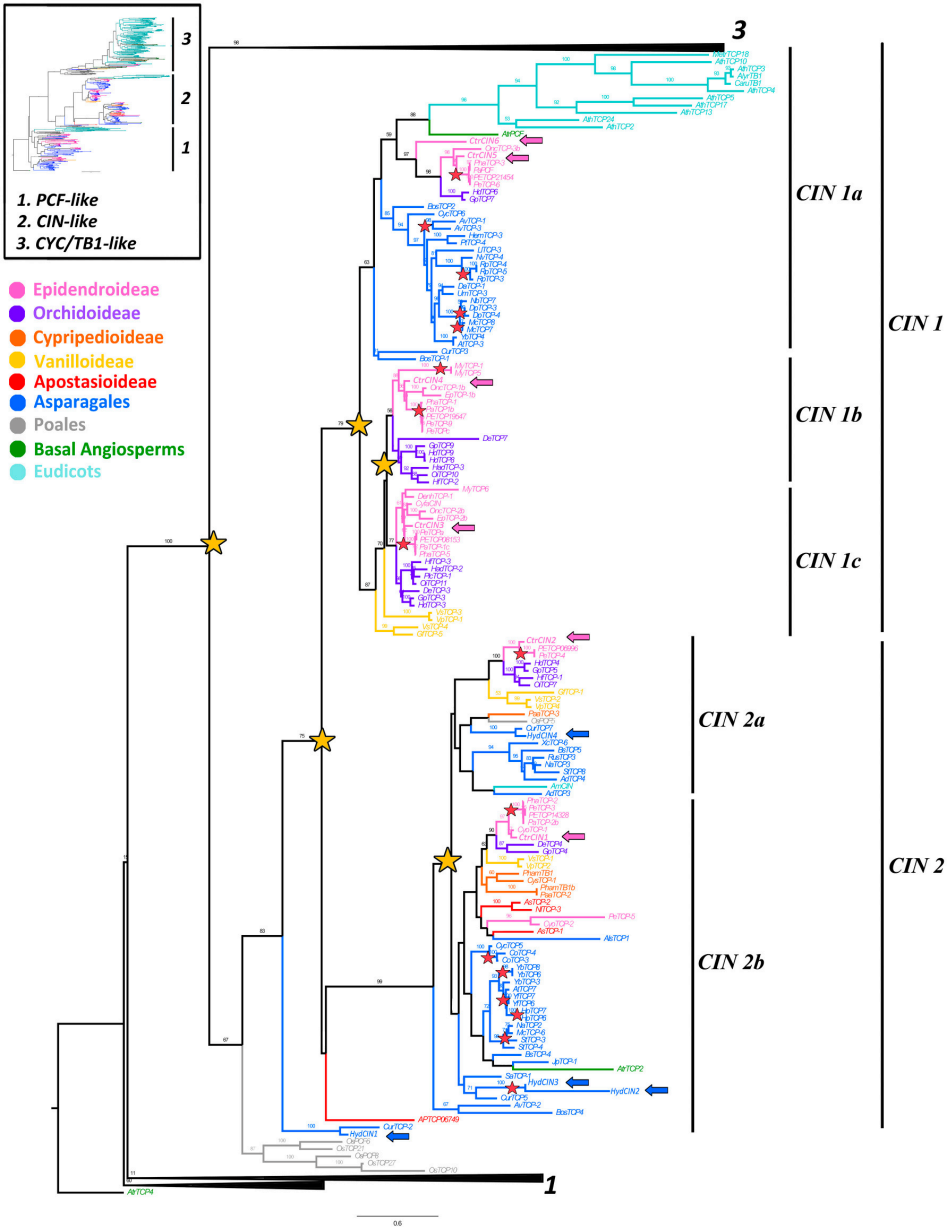
**ML analysis of ***CIN-like*** genes**. Overview (upper left), summary tree as in Figure 1. To the right, ML phylogenetic analysis of *TCP* genes expanded to show the *CIN-like* clade (2). Yellow stars indicate large scale duplication events: one prior to the diversification of angiosperms yielding clades CIN1 and CIN2, two before the radiation of Asparagales and one prior to the origin of Epidendroideae and Orchidoideae; red stars indicate species-specific duplication events; blue and pink arrows indicate *H. decumbens* and *C. trianae* homologs, respectively. Branch and taxa colors correspond to those in the conventions to the left. BS values ≥ 50 are shown.

The *CIN-like* ML analysis shows a duplication (BS = 75) that predates the diversification of angiosperms resulting in the *CIN1* (BS = 79) and *CIN2* clades (BS = 99) (Figure 3). This is confirmed by the position of the two *A. trichopoda CIN* paralogs, *AtrPCF* and *AtrTCP2*, each in its own clade (Figure 3). Additional support for this early duplication is found in the topology yielded by a second complementary analysis that includes 11 Solanaceae *CIN* homologs, where *CIN1* and *CIN2* clades have monocot and core eudicot representatives and, at least *CIN2* is well supported (BS = 93) (Supplementary Figure 2). The *CIN1* clade has undergone at least two additional duplications resulting in the *CIN1a-c* clades. It is likely that the duplication resulting in *CIN1a* and *CIN1b*/*c* occurred exclusively in monocots, although the exact timing is unclear. The other duplication resulting in *CIN1b* and *CIN1c* appears to be Orchidaceae-specific, prior to the diversification of Orchidoideae and Epidendroideae (Figure 3). On the other hand, the *CIN2* clade underwent an independent duplication predating the diversification of Asparagales, resulting in *CIN2a* and *CIN2b* subclades. Intraspecific duplications were identified in *Hesperaloe, Disporopsis, Maianthemum, Rhodophiaia, Sansevieria* and *Yucca*, (Figure 3). Poales *CIN* homologs form a clade, with a low BS, in the first analysis, with the exception of *OsPCF5* clustered with *AmCIN* (Figure 3). However, our second analysis shows two Poales clades nested in each angiosperm paralogous *CIN1* and *CIN2* clades (with low BS), suggesting that the two Poales clades likely resulted from the angiosperm *CIN1/2* duplication (Supplementary Figure 2).

CIN-like sequences show high degree of conservation at the N-flank of the TCP domain (Figure 2). The only changes with respect to the key aminoacids in the bHLH domain in CYC proteins are at the second helix where the LxxLL motif shifts to V/IxxLL (Figure 2). Toward the 3′ end of the TCP domain proteins are highly variable, except for motifs 13, 14, 16–18 and 21, reported also by De Paolo et al. (2015) (Supplementary Figure 1). The R domain (motif 11) in CIN proteins is only present in the *CIN1a* clade and *O. sativa* homologs *OsTCP21, OSTCP8* and *OsTCP10*. Motif 14, which corresponds to the *miR319* binding site is present in most *CIN-like* sequences. The *miR319* binding motif is lacking in *HydCIN2, HydCIN3, OsTCP21, OsTCP27*, and *OsTCP10*. All *CIN1* subclade sequences share motifs 13, 17, 19, 20 21, and 25; the *CIN1a* subclade shares motifs 11, 28, 29, 34 and 35, while the *CIN1b* subclade shares motifs 15, 23, 26, and 30. Synapomorphies for the *CIN2* subclade include motifs 16, 18, 22, 24 and only motif 27 is exclusive to Orchidaceae. Finally, *CIN2b* homologs share motifs 32 and 33. The most divergent *C. trianae* sequence is *CtrCIN6*, which only has the motifs 1, 2, 3, 11, 13, 14, 17, and 21.

### *PCF-like* gene evolution

Our analysis recovered 129 *PCF-like* homologs (Supplementary Table 1). Similarly to *CIN-like* genes most sampling is concentrated in Asparagales, thus 53 homologs belong to 35 species of non-Orchidaceae Asparagales and 52 sequences correspond to Orchidaceae. *H. decumbens* has 10 *PCF-like* copies (*HydPCF1–HydPCF10*). Sampling in Orchidaceae includes one homolog from one Vanilloideae species, 14 homologs from seven Orchidoideae species and 37 homologs from 11 Epidendroideae species. We recovered 11 *PCF-like* homologs from *C. trianae* (*CtrPCF1–CtrPCF11*). Sampling in monocots outside Asparagales include 10 homologs from *O. sativa* (Poales) and sequences that are not monocots are restricted to one homolog from *A. trichopoda* and 13 homologs from *A. thaliana*.

**Figure 4:**
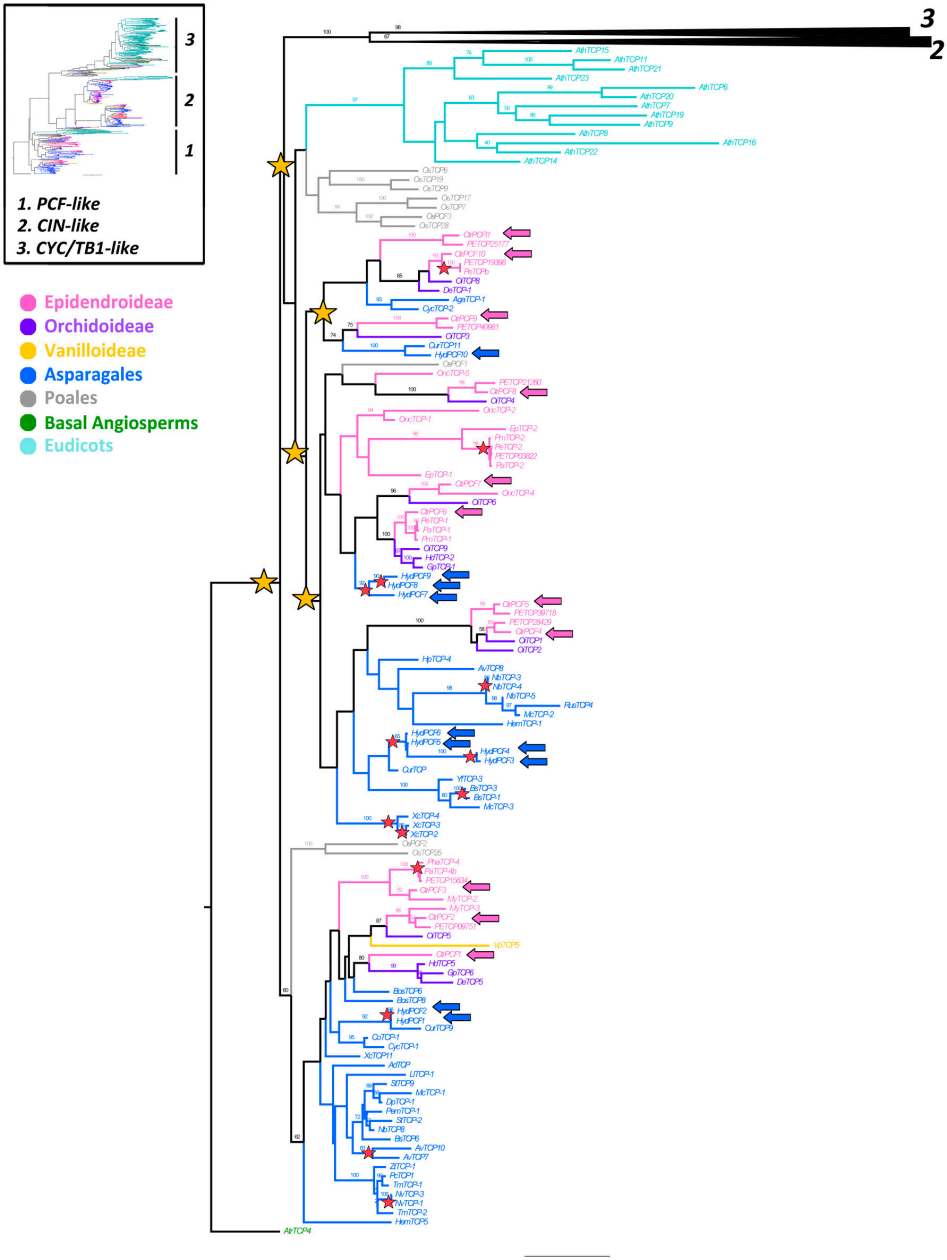
**ML analysis of ***PCF-like*** genes**. Overview (upper left) summary tree as in Figure 1. To the right, ML phylogenetic analysis of *TCP* genes expanded to show the *PCF-like* clade (1); yellow stars indicate large scale duplication events at least three before the diversification of Asparagales; red stars indicate species-specific duplication events; blue and pink arrows indicate *H. decumbens* and *C. trianae* homologs, respectively. Branch and taxa colors correspond to those in the conventions to the left. BS values ≥ 50 are shown.

Our analysis detected at least five duplication events of *PCF-like* prior to the diversification of Asparagales, however support is low for all clades (BS = < 50) (Figure 4). In addition, our complementary analysis including 18 Solanaceae *PCF-like* genes also shows support for at least two rounds of core eudicot specific *PCF-like* duplications (Supplementary Figure 2).

PCF-like sequences exhibit the shortest basic motif in TCP proteins, with a deletion in the bipartite NLS between positions 10 and 13 (Figure 2; Cubas et al., 1999). The TCP domain shows little conservation in comparison to the TCP II class (CYC and CIN) proteins. For instance, there is a four amino acid deletion in the middle of the basic motif, and only 12 out of the 23 amino acids characterized in both helices and the loop are conserved (Figure 2). Additionally our MEME analysis show that PCF-like proteins do not have an R domain (motif 11), nor a target sequence for *miR319* (motif 14) (Supplementary Figure 1).

### Expression of *TCP-like* homologs from *Hypoxis decumbens* and *Cattleya trianae*

**Figure 5:**
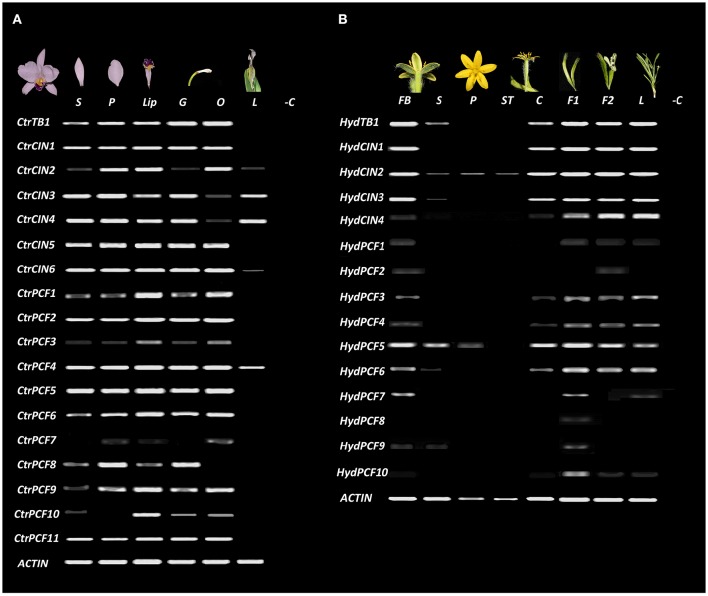
*****TCP-like*** expression analysis in Asparagales**. *ACTIN* was used as positive control. **(A)** Expression of all isolated *TCP-like* transcription factors in dissected floral organs and leaves of *C. trianae* (Orchidaceae). **(B)** Expression of all isolated *TCP-like* transcription factors in dissected floral organs, fruits and leaves of *H. decumbens* (Hypoxidaceae). BF, flower bud; C, carpels; F1, immature fruit; F2, mature fruit; G, gynostemium; L, Leaves; Lip, lip; O, Ovary; P, petals; S, sepals; ST, stamens. −C Indicates the amplification reaction of PCR without cDNA (negative control).

In order to hypothesize functional roles for the Asparagales *TCP-like* homologs, the expression patterns of all homologs isolated from transcriptomic analysis in *H. decumbens* and *C. trianae* were evaluated (Figure 5). Although in both species we were able to dissect floral organs in preanthesis and young leaves, we were not able to find fruits of *C. trianae*, thus only young and old fruits of *H. decumbens* were included in the expression study.

The *CYC/TB1-like* homologs have very different expression patterns in *C. trianae* and *H. decumbens*. *HydTB1* is expressed in the floral bud, sepals, carpels, young and mature fruits and leaves, whereas *CtrTB1* is expressed in all floral whorls and is not expressed in leaves (Figure 5). *CIN-like* homologs also exhibit different expression patterns in *C. trianae* and *H. decumbens*. *HydCIN1, HydCIN2* and *HydCIN3* are expressed in the floral bud, carpels, fruits and leaves. Only *HydCIN2* expression is extended to sepals, petals and stamens at very low levels. Interestingly both *HydCIN2* and *HydCIN3* lack the *miR319* binding site. *HydCIN4* expression is restricted to fruits and leaves. *CtrCIN1, CtrCIN2, CtrCIN5*, and *CtrCIN6* are expressed in sepals, petals, lip, gynostemium, and ovary, although, *CtrCIN2* expression in sepals and gynostemium occurs at low levels. *CtrCIN3* and *CtrCIN4* have similar expression patterns to *CtrCIN1*, however, they are poorly expressed in the ovary and are the only *CIN-like* homologs that extend their expression to leaves.

Similar to CYC/TB1-like and CIN-like genes, the expression of PCF-like homologs varies dramatically between *C. trianae* and *H. decumbens* (Figure 5). *CtrPCF1, CtrPCF2, CtrPCF3, CtrPCF4, CtrPCF5, CtrPCF6, CtrPCF9*, and *CtrPCF11* are expressed in all floral organs and the only copy with expression in leaves is *CtrPCF4*. *CtrPCF3* has low expression in sepals and petals. *CtrPCF7* is only expressed in petals and ovary. Finally, *CtrPCF10* has expression in all floral whorls except in petals. In *H. decumbens* only *HydPCF3, HydPCF4, HydPCF5*, and *HydPCF6* are expressed in the floral buds, carpels, fruits and leaves, although *HydPCF3* and *HydPCF4* have low expression in carpels. *HydPCF5* is also expressed in the perianth with higher expression in sepals than in petals. *HydPCF7* has restricted expression to the floral buds and the young fruits. *HydPCF9* and *HydPCF10* are only expressed in young fruits and the remaining copies (*HydPCF1, HydPCF2*, and *HydPCF8*) are only expressed at very low levels in the floral buds.

## Discussion

Most functional studies on *TCP* genes have focused on identifying their contribution to floral symmetry and plant architecture, as expected by the functions of the canonical *CYC* and *DICH* from *A. maju*s and *AtTCP1* from *A. thaliana* respectively (Luo et al., 1996, 1999; Costa et al., 2005). Studies on the evolution of TCP transcription factors have concentrated on core eudicots and particularly on *CYC2* homologs (Damerval and Manuel, 2003; Howarth and Donoghue, 2006; Preston and Hileman, 2009; Martín-Trillo and Cubas, 2010; Mondragón-Palomino and Trontin, 2011; Sarvepalli and Nath, 2011; Danisman et al., 2012, 2013; Uberti-Manassero et al., 2012; Aguilar-Martínez and Sinha, 2013; Das Gupta et al., 2014; Lin et al., 2016). Our matrix includes sampling from the *Phalaenopsis* genome as well as all transcriptomes available for Asparagales. The phylogenetic analysis made with the full-length coding sequences, allowed us to identify a number of large scale as well as local *TCP* gene duplications and changes in protein sequences linked to these duplications. Moreover, this is the first large scale analysis of *CIN-like* and *PCF-like* genes. We are able to report a comparative expression pattern in two Asparagales species, representing the two floral groundplans in the order, and present hypotheses on the putative role of these genes in floral patterning in representative Asparagales.

### Asparagales *CYC/TB1* homologs are found predominantly as single copies and have divergent expression patterns in *Hypoxis decumbens* and *Cattleya trianae*

Our study detected a single copy *CYC/TB1* gene in each of the species investigated in the Asparagales. The Asparagales *CYC/TB1* homologs fall outside of the Poales (TB1/REP clades) or Commelinales identified local duplications, described before and recovered here (Doebley et al., 1995, 1997; Yuan et al., 2009; Mondragón-Palomino and Trontin, 2011). The tree topology suggests that independent duplications have occurred in *CYC-like* genes in the monocots (Yuan et al., 2009; Mondragón-Palomino and Trontin, 2011; Hileman, 2014). Species-specific duplications in Asparagales were found only in *Phalaenopsis* (Orchidaceae). Moreover, expression data of *CYC/TB1* genes in Asparagales show remarkable differences between *H. decumbens* and *C. trianae*, even though sampled organs correspond to fairly well developed tissues. *CtrTB1* is expressed homogeneously in all floral whorls while *HydTB1* is expressed predominantly in carpels, fruits and leaves (Figure 5). Homogeneous expression of *CYC-like* genes in dorsal and ventral floral organs in *C. trianae* suggest that *CtrTB1* is likely not playing important roles in maintenance of bilateral symmetry in orchid flowers (see also Horn et al., 2015). However, only pre-anthethic floral buds were sampled and earlier stages are needed to test whether *CtrTB1* can be contributing to the establishment of bilateral symmetry in the flower primordia. Comparative expression studies of *CYC/TB1-like* genes in other Orchidaceae point to significant variations. For instance, while the *O. italica* homolog *OitaTB1* is only expressed in leaves (De Paolo et al., 2015), two *CYC/TB1* genes in *P. equestris* do exhibit differential dorsiventral expression in the floral bud suggesting species specific roles in bilateral symmetry establishment (Lin et al., 2016).

Expression of *CYC/TB1* genes in Orchidaceae contrasts with studies in Commelinales, Zingiberales, and Alstroemeriaceae that show differential dorsiventral expression of *CYC/TB1* genes and hence support convergent recruitment of *CYC* homologs in the acquisition of bilateral symmetry in different monocots (Bartlett and Specht, 2011; Preston and Hileman, 2012; Hoshino et al., 2014). Within core eudicots, only the *CYC2/TCP1* clade members have been linked to shifts toward bilateral floral symmetry. This has been extensively documented for *CYC* and *DICH* in *A. majus* (recent paralogs within the *CYC2/TCP1* clade) which are expressed in the dorsal regions of the floral meristems and negatively regulate cell proliferation (Luo et al., 1996, 1999). However, many other *CYC2* orthologs have been shown to control bilateral symmetry in Asterales, Brassicales, Dipsacales, Fabales, Lamiales, and Malpighiales (Busch and Zachgo, 2007; Gao et al., 2008; Preston et al., 2009; Wang et al., 2010; Zhang et al., 2010, 2013; Howarth et al., 2011; Tähtiharju et al., 2012; Yang et al., 2012; Ma et al., 2016).

Recruitment of CYC2 homologs in bilateral symmetry is likely facilitated by conserved protein-protein interactions mediated by the LxxLL motif (Heery et al., 1997; Damerval and Manuel, 2003; Reeves and Olmstead, 2003; Howarth and Donoghue, 2006; Li et al., 2009; Preston et al., 2009; Tähtiharju et al., 2012; Parapunova et al., 2014; Ma et al., 2016). If so, it is possible that different CYC-like proteins can form specific homo- and heterodimers, or even have unique partners, and thus protein motifs can provide clues to protein affinity and functional specificity (Kosugi and Ohashi, 2002). Our MEME analysis rescues motifs 6, 9 and 12, shared only between *P. equestris* PETCP11715 and the *A. majus AmCYC* and *AmDICH*, which are not present in other orchid CYC/TB1 proteins (i.e., *C. trianae* and *O. italica*) and hence putatively involved in establishing early bilateral floral symmetry in some Orchidaceae species (Supplementary Figure 1). When full length CtrTB1 is compared to the three *P. equestris* CYC/TB1 proteins very little similarity is detected (PETCP11715–0.38; PETCP06750–0.22; PETCP06750–0.24); for instance, CtrTB1 lacks motif 11 (R-domain). Nevertheless, the TCP domain is extremely conserved (0.91, 0.80, and 0.84 respectively). These results suggest that besides the key bHLH amino acids that target conserved genes, there are likely important motifs in the flanking regions allowing unique interactions and downstream partners in each species.

Conversely, the expression of *HydTB1* in *H. decumbens* is indicative of exclusive roles in carpels, fruits and leaves. This data suggests that *HydTB1* may have similar roles to other *CYC* genes that do not participate in establishing floral bilateral symmetry. Such is the case of the *A. thaliana, AtTCP1*, which promotes shoot growth and regulates leaf lamina size (Costa et al., 2005; Guo et al., 2010; Koyama et al., 2010b). To date less attention has been given to the putative role of *CYC* genes in the development of leaves, carpels and fruits, despite the fact that expression has been detected in these organs in other core eudicots. This is the case for *CYC* homologs in *Citrullus, Gerbera, Gossypium, Lotus, Solanum*, and some Papaveraceae that have been detected in seedlings, young leaves, and immature fruits (Damerval et al., 2007; Wang et al., 2010; Parapunova et al., 2014; Ma et al., 2016; Shi et al., 2016).

### *CIN-like* genes have undergone numerous duplications in angiosperms, monocots and orchidaceae and show broad expression patterns in *Cattleya trianae* when compared to *Hypoxis decumbens*

Here we show the first comprehensive phylogenetic analysis of *CIN-like* genes. Our results point to the occurrence of at least one duplication event predating angiosperm diversification, at least one duplication occurring prior to the origin of the Asparagales and one specific duplication prior to the diversification of Orchidoideae and Epidendroideae (Figure 3, Supplementary Figure 2; Floyd and Bowman, 2007; Martín-Trillo and Cubas, 2010). By comparison to *CYC* genes, *CIN* genes functional characterization is restricted to model species only. The canonical *CINCINNATA* in *A. majus* has dual roles in limiting the growth of leaf margins while promoting epidermal cell differentiation in petals (Nath et al., 2003; Crawford et al., 2004). The *cin* mutant in *A. majus*, as well as the *tcp4* mutant in *A. thaliana*, exhibit curly leaves as a result of excessive growth in leaf margins (Crawford et al., 2004; Koyama et al., 2010a). *CIN* regulates leaf shape through direct or indirect negative regulation of the boundary *CUP-SHAPED COTYLEDON (CUC)* genes, likely via the activation of *ASSYMETRIC LEAVES 1* (*AS1*), *miR164, INDOLE-3-ACETIC ACID3/SHORT HYPOCOTYL2 (IAA3/SHY2)*, and *SMALL AUXIN UP RNA (SAUR)* (Koyama et al., 2007, 2010a). *CIN* also controls leaf development through the regulation of cell proliferation by activating *miR396, CYCLIN-DEPENDENT KINASE INHIBITOR/KIP RELATED PROTEIN 1 (ICK1/KRP1)* and jasmonate biosynthesis (Schommer et al., 2014). Similarly, *AtTCP4* regulates the transition between cell proliferation and differentiation, controlling cytokinin and auxin receptors (Efroni et al., 2013; Das Gupta et al., 2014). *AtTCP4* also controls leaf senescence, maintains petal growth, and regulates early embryo development and seed viability, and finally it regulates *jasmonic acid (JA)* biosynthesis by the activation of *LIPOXIGENASE2 (LOX2)* (Schommer et al., 2008; Nag et al., 2009; Sarvepalli and Nath, 2011; Danisman et al., 2012). Leaf patterning is also controlled by *CIN-like* homologs in tomato and rice (Ori et al., 2007; Yang et al., 2013; Zhou et al., 2013; Ballester et al., 2015).

In addition to the roles of *CIN* genes in leaf patterning, other functions in carpel and fruit development have been identified. *AtTCP2* and *AtTCP3* are known to activate *NGATHA* genes that regulate carpel apical patterning. *NGA* orthologs from *A. thaliana*, rice, tomato and bean have conserved putative TCP binding site suggesting that this regulation is conserved in monocots and dicots (Ballester et al., 2015). Moreover, the *tcp3* mutant has shorter siliques with a crinkled surface (Koyama et al., 2007; Ballester et al., 2015). Additionally, expression analyses in different Solanaceae species suggest an important contribution of these genes to fruit development and maturation, as they are downstream targets of key ripening regulators including *RIPENING INHIBITOR* (*RIN*), *COLORLESS NON-RIPENING* (*CNR*) and *SlAP2a* (Supplementary Figure 3; Crawford et al., 2004; Parapunova et al., 2014). More recently *PeCIN8*, a *P. equestris CIN-like* homolog, was shown to be broadly expressed and to have roles in leaf cell proliferation, determining the fruit final size and controlling proper embryo and ovule development (Lin et al., 2016). In summary, *CIN-like* genes are pleiotropic regulators of cell division and differentiation in leaves, petals, carpels, ovules, fruits and seeds across angiosperms.

This study identified significant changes in the helix I residues and the loop between Orchidaceae sequences and other Asparagales CIN-like proteins (Figure 2). For instance, a number of motifs involved in protein interaction, including the R domain characteristic of class II TCP proteins were only identified in the CIN1a clade, but are absent in all other paralogous clades (Supplementary Figure 1, Cubas et al., 1999; Damerval and Manuel, 2003). Here we have also identified conserved motifs like the *miR319* binding site, previously reported in *CIN-like* homologs from *O. italica* (Nag et al., 2009; De Paolo et al., 2015), for all *CIN-like* sequences in Asparagales. This indicates, that Asparagales homologs are also regulated by *miR319*, similar to *AtTCP4*, and other *CIN-like Arabidopsis* paralogs, including *AtTCP2/3/10* and *24* (Palatnik et al., 2003; Koyama et al., 2007; Schommer et al., 2008, 2012; Koyama et al., 2010a; Danisman et al., 2012).

In comparison with reported functional data from model eudicots and monocots, the *CIN-like* differential expression observed in *C. trianae* and *H. decumbens* homologs points to three testable hypotheses. (1) While in *H. decumbens* all *CIN-like* genes are expressed in young leaves and may play roles in leaf development, only two of the six paralogs identified in *C. trianae* (*CtrCIN3*/*4*) are likely involved in cell division and differentiation during leaf development, similar to the *P. equestris PeCIN8* (Lin et al., 2016). (2) While *CIN-like* genes are likely playing key roles in both perianth and fertile organs development and growth in *C. trianae*, their contribution to perianth development and growth is less clear in *H. decumbens*, perhaps only with *HydCIN2* involved in cell proliferation in the perianth. (3) In both species *CIN-like* genes are strongly expressed in carpels and fruits, suggesting that their role in carpel patterning, ovule development as well as fruit maturation is likely conserved in Asparagales. Nevertheless, mRNA expression data for all *CIN-like* genes must be interpreted with caution given the putative conserved *miR139* postranscriptional regulation.

### *PCF-like* genes are extensively duplicated and have overlapping expression patterns with *CIN-like* genes in asparagales

Our results on the evolution of *PCF-like* genes points to numerous duplication events within Asparagales in comparison to the *CYC-like* and the *CIN-like* clades. In addition, the topology recovered suggests that *PCF-like* gene duplications in monocots are independent from the one that occurred in core eudicots (Figure 4, Supplementary Figure 2). Functional data available for *PCF-like* genes suggest redundancy with *CIN-like* genes (Aguilar-Martínez and Sinha, 2013; Danisman et al., 2013). For instance, both *PCF-like* and *CIN-like* genes control leaf development through regulation of *LOX2*. However, while *AtTCP20 (PCF-like)* inhibits, *AtTCP4 (CIN-like)* induces the expression of *LOX2* (Danisman et al., 2012). *PCF-like* genes are also involved in the regulation of circadian clock genes, that is the case of *CCA1 Hiking Expedition CHE (AtTCP21)* (Pruneda-Paz et al., 2009; Giraud et al., 2010). In addition dimers formed between AtTCP15 and other TCP-like proteins (AtTCP2, AtTCP3, AtTCP11) are known to regulate circadian cycles, cell proliferation in floral organs and leaves, and to promote seed germination (Koyama et al., 2007; Kieffer et al., 2011; Resentini et al., 2015). *PCF-like* genes are also expressed in ovule, seed and fruit development in *Solanum lycopersicum, Solanum tuberosum, O. sativa* and in *P. equestris*, suggesting that they mediate cell proliferation in the carpel to fruit transition in both eudicots and monocots (Supplementary Figure 3; Kosugi and Ohashi, 1997; Yao et al., 2007; Parapunova et al., 2014; Lin et al., 2016).

Expression detected here of *PCF-like* homologs in *C. trianae* and *H. decumbens* show broad expression of most paralogs in *C. trianae* (except *CtrPCF7*) contrasting with a restricted expression of most paralogs in *H. decumbens* (except *HydPCF5*; Figure 5). Such expression patterns are in accordance with all putative functions identified in other monocots and in core eudicots including, but not restricted to, cell proliferation control in leaves, carpels, ovules, fruits and seeds (Lin et al., 2016). All data available point to pleiotropic roles of *TCP-like* genes with a high degree of redundancy among paralogs. Interestingly, when compared to *CYC-like and CIN-like* genes, the *PCF-like* gene *HydPCF5* in *H. decumbens* is more likely to be playing cell proliferation roles in the perianth.

In conclusion, the Asparagales, unlike core eudicot model plants, have a reduction in the number of *CYC-like* homologs and an increase of *CIN-like* and *PCF-like* copies. Characteristic key amino acids in the bHLH domain, flanking motifs and binding sites are for the most part conserved in Asparagales CIN-like and PCF like proteins suggesting similar conserved mechanisms of post-transcriptional regulation and interacting partners. The most notorious exception to this is the lack of a *miR319* binding site in *HydCIN2/3*. Nevertheless, the CYC-like proteins in Asparagales seem to be poorly expressed and have undergone important shifts in protein domains, suggesting changes in regulation as well as in protein-protein interactions. The orchid included in this study has similar numbers of TCP copies when compared to Asparagales with radially symmetrical flowers, and only the Epidendrioideae and Orchidoideae seem to have an additional *CIN* paralog lacking in other Orchidaceae and Asparagales. Expression data suggests different roles of *TCP-like* genes in *C. trianae* and *H. decumbens*, pointing to: (1) a possible decoupling of *TB1* homologs from bilateral symmetry in *C. trianae*; (2) conserved roles of *CIN-like* and *PCF-like* genes in the control of cell proliferation in carpels, ovules and fruits in both species; and (3) preferential leaf expression of *CIN-like* and *PCF-like* genes in *H. decumbens* and perianth expression in *C. trianae*. Here we have performed the first large scale analysis of *TCP* genes in Asparagales which will provide a platform for in depth comparative expression analyses as well as much needed functional studies of these genes in emerging model orchids.

## Author contributions

YM and NP planned and designed the research. YM and NP conducted fieldwork. YM, JA, and NP performed experiments. YM, JA, and NP analyzed the data and wrote and approved the final version of the manuscript. All authors read and approved the final manuscript.

### Conflict of interest statement

The authors declare that the research was conducted in the absence of any commercial or financial relationships that could be construed as a potential conflict of interest.

## Supplementary material

Supplementary Figure 1 **Conserved motifs in and Orchidaceae and non-Orchidaceae Asparagales TCP-like proteins**. Model core eudicots and monocots used as reference include *Arabidopsis thaliana, Antirrhinum majus*, and *Oryza sativa*. Motifs 1, 2, and 3 correspond to the conserved TCP domain. Motif 11 indicates the characteristic R domain in Class II TCP-like genes. Motif 14 corresponds to the *miR319* binding site in *CIN-like* genes.

Supplementary Figure 2 **ML analysis of ***TCP-like*** genes with extended Solanaceae sampling**. ML phylogenetic analysis of *TCP-like* genes with reduced sampling including only model organisms like *Arabidopsis thaliana, Oryza sativa, Solanum lycopersicum, Solanum tuberosum*, two Orchidaceae species, *Cattleya trianae, Orchis italica*, one non-Orchidaceae Asparagales, *Hypoxis decumbens* and the early diverging angiosperm *Amborella trichopoda*. Yellow stars indicate large scale duplication events. TCP major clades are labeled to the right. Branch and taxa colors correspond to those in the conventions. BS values ≥ 50 are shown.

Supplementary Figure 3 **Expression patterns of TCP-like homologs in ***Arabidopsis thaliana*** (Brassicaceae), ***Solanum lycopersicum*** and ***Solanum tuberosum*** (Solanaceae). (A)** *Arabidopsis thaliana* AthTCP24 (CIN-like). **(B)** *Arabidopsis thaliana* AthTCP20 (PCF-like). **(C)** Solanum lycopersicum SlyTCP24 (CIN-like). **(D)** *Solanum lycopersicum* SlyTCP11 (PCF-like). **(E)** *Solanum tuberosum* StuTCP2 (CIN-like). **(F)** *Solanum tuberosum* StuTCP11 (PCF-like). Expression patterns show here were selected for the genes that showed the broader, most comprehensive expression patterns after comparing expression for all paralogs, and are used to summarize putative expression patterns for each gene clade. Expression levels vary in intensity between different copies of CIN-like and PCF-like. Taken from the eFP browser, last accessed Sep 5/2016 (http://bar.utoronto.ca/efp_tomato/cgi-bin/efpWeb.cgi and http://bar.utoronto.ca/efp_potato/cgi-bin/efpWeb.cgi).

Supplementary Table 1 **List of sequences used in this study**.

Supplementary Table 2 **Primers used for ***TCP-like gene*** expression analyses**.

## References

[B1] Aguilar-Martínez J. A. Sinha N. (2013). Analysis of the role of Arabidopsis class I *TCP* genes *AtTCP7, AtTCP8, AtTCP22*, and *AtTCP23* in leaf development. Front. Plant Sci. 4:406. 10.3389/fpls.2013.00406 24137171 PMC3797442

[B2] Almeida J. Rocheta M. Galego L. (1997). Genetic control of flower shape in *Antirrhinum majus*. Development 124, 1387–1392. 9118809 10.1242/dev.124.7.1387

[B3] Altschul S. F. Gish W. Miller W. Myers E. W. Lipman D. J. (1990). Basic local alignment search tool. J. Mol. Biol. 215, 403–410. 10.1016/S0022-2836(05)80360-2 2231712

[B4] Bailey T. L. Williams N. Misleh C. Li W. W. (2006). MEME: discovering and analyzing DNA and protein sequence motifs. Nucleic Acids Res. 34, W369–W373. 10.1093/nar/gkl198 16845028 PMC1538909

[B5] Ballester P. Navarrete-Gómez M. Carbonero P. Oñate-Sánchez L. Ferrándiz C. (2015). Leaf expansion in Arabidopsis is controlled by a TCP-NGA regulatory module likely conserved in distantly related species. Physiol. Plant 155, 21–32. 10.1111/ppl.12327 25625546

[B6] Bartlett M. E. Specht C. D. (2011). Changes in expression pattern of the *TEOSINTE BRANCHED1*- like genes in the Zingiberales provide a mechanism for evolutionary shifts in symmetry across the order. Am. J. Bot. 98, 227–243. 10.3732/ajb.1000246 21613112

[B7] Broholm S. (2009). The Role of MADS and TCP Transcription Factors in Gerbera Hybrida Flower Development. Available online at: https://helda.helsinki.fi/handle/10138/22335

[B8] Busch A. Zachgo S. (2007). Control of corolla monosymmetry in the *Brassicaceae Iberis* amara. Proc. Natl. Acad. Sci. U.S.A. 104, 16714–16719. 10.1073/pnas.0705338104 17940055 PMC2034219

[B9] Chase M. W. Cameron K. M. Freudenstein J. V. Pridgeon A. M. Salazar G. van den Berg C. (2015). An updated classification of Orchidaceae. Bot. J. Linn. Soc. 177, 151–174. 10.1111/boj.12234

[B10] Chase M. W. Christenhusz M. J. M. Fay M. F. Byng J. W. Judd W. S. Soltis D. E. (2016). An update of the Angiosperm Phylogeny Group classification for the orders and families of flowering plants: APG IV. Bot. J. Linn. Soc. 181, 1–20. 10.1111/boj.12385

[B11] Chase M. W. Reveal J. L. Fay M. F. (2009). A subfamilial classification for the expanded asparagalean families Amaryllidaceae, Asparagaceae and Xanthorrhoeaceae. Bot. J. Linn. Soc. 161, 132–136. 10.1111/j.1095-8339.2009.00999.x

[B12] Chen S. Kim D. K. Chase M. W. Kim J. H. (2013). Networks in a large-scale phylogenetic analysis: reconstructing evolutionary history of Asparagales (Lilianae) based on four plastid genes. PLoS ONE 8:e59472. 10.1371/journal.pone.0059472 23544071 PMC3605904

[B13] Citerne H. L. Le Guilloux M. Sannier J. Nadot S. Damerval C. (2013). Combining phylogenetic and syntenic analyses for understanding the evolution of TCP ECE genes in eudicots. PLoS ONE 8:e74803. 10.1371/journal.pone.0074803 24019982 PMC3760840

[B14] Corley S. B. Carpenter R. Copsey L. Coen E. (2005). Floral asymmetry involves an interplay between TCP and MYB transcription factors in Antirrhinum. Proc. Natl. Acad. Sci. U.S.A. 102, 5068–5073. 10.1073/pnas.0501340102 15790677 PMC555980

[B15] Costa M. M. R. Fox S. Hanna A. I. Baxter C. Coen E. (2005). Evolution of regulatory interactions controlling floral asymmetry. Development 132, 5093–5101. 10.1242/dev.02085 16236768

[B16] Crawford B. C. W. Nath U. Carpenter R. Coen E. S. (2004). CINCINNATA controls both cell differentiation and growth in petal lobes and leaves of Antirrhinum. Plant Physiol. 135, 244–253. 10.1104/pp.103.036368 15122032 PMC429364

[B17] Cubas P. Lauter N. Doebley J. Coen E. (1999). The TCP domain: a motif found in proteins regulating plant growth and development. Plant J. 18, 215–222. 10.1046/j.1365-313X.1999.00444.x 10363373

[B18] Damerval C. Citerne H. Le Guilloux M. Domenichini S. Dutheil J. De Craene L. R.. (2013). Asymmetric morphogenetic cues along the transverse plane: shift from disymmetry to zygomorphy in the flower of fumarioideae. Am. J. Bot. 100, 391–402. 10.3732/ajb.1200376 23378492

[B19] Damerval C. Le Guilloux M. Jager M. Charon C. (2007). Diversity and evolution of *CYCLOIDEA-like* TCP genes in relation to flower development in Papaveraceae. Plant Physiol. 143, 759–772. 10.1104/pp.106.090324 17189327 PMC1803737

[B20] Damerval C. Manuel M. (2003). Independent evolution of Cycloidea-like sequences in several angiosperm taxa. Comptes Rendus Palevol 2, 241–250. 10.1016/S1631-0683(03)00031-9

[B21] Danisman S. van der Wal F. Dhondt S. Waites R. de Folter S. Bimbo A.. (2012). Arabidopsis class I and class II TCP transcription factors regulate jasmonic acid metabolism and leaf development antagonistically. Plant Physiol. 159, 1511–1523. 10.1104/pp.112.200303 22718775 PMC3425195

[B22] Danisman S. Van Dijk A. D. J. Bimbo A. van der Wal F. Hennig L. De Folter S.. (2013). Analysis of functional redundancies within the Arabidopsis TCP transcription factor family. J. Exp. Bot. 64, 5673–5685. 10.1093/jxb/ert337 24129704 PMC3871820

[B23] Das Gupta M. Aggarwal P. Nath U. (2014). CINCINNATA in *Antirrhinum majus* directly modulates genes involved in cytokinin and auxin signaling. New Phytol. 204, 901–912. 10.1111/nph.12963 25109749

[B24] De Paolo S. Gaudio L. Aceto S. (2015). Analysis of the TCP genes expressed in the inflorescence of the orchid *Orchis italica*. Sci. Rep. 5:16265. 10.1038/srep16265 26531864 PMC4632031

[B25] Doebley J. Stec A. Gustus C. (1995). teosinte branched1 and the origin of maize: evidence for epistasis and the evolution of dominance. Genetics 141, 333–346. 8536981 10.1093/genetics/141.1.333 PMC1206731

[B26] Doebley J. Stec A. Hubbard L. (1997). The evolution of apical dominance in maize. Nature 386, 485–488. 10.1038/386485a0 9087405

[B27] Efroni I. Han S. K. Kim H. J. Wu M. F. Steiner E. Birnbaum K. D.. (2013). Regulation of leaf maturation by chromatin-mediated modulation of cytokinin responses. Dev. Cell 24, 438–445. 10.1016/j.devcel.2013.01.019 23449474 PMC3994294

[B28] Endress P. K. (2016). Development and evolution of extreme synorganization in angiosperm flowers and diversity: a comparison of Apocynaceae and Orchidaceae. *Ann*. Bot. 117, 749–767. 10.1093/aob/mcv119 PMC4845794 26292994

[B29] Floyd S. K. Bowman J. L. (2007). The ancestral developmental tool kit of land plants. Int. J. Plant Sci. Spec. Issue Discern. Homol. Gene Expr. 168, 1–35. 10.1086/509079

[B30] Galego L. Almeida J. (2002). Role of *DIVARICATA* in the control of dorsoventral asymmetry in *Antirrhinum* flowers. Genes Dev. 16, 880–891. 10.1101/gad.221002 11937495 PMC186332

[B31] Gao Q. Tao J. H. Yan D. Wang Y. Z. Li Z. Y. (2008). Expression differentiation of *CYC*-like floral symmetry genes correlated with their protein sequence divergence in *Chirita heterotricha* (Gesneriaceae). Dev. Genes Evol. 218, 341–351. 10.1007/s00427-008-0227-y 18592267

[B32] Giraud E. Ng S. Carrie C. Duncan O. Low J. Lee C. P.. (2010). TCP transcription factors link the regulation of genes encoding mitochondrial proteins with the circadian clock in *Arabidopsis thaliana*. Plant Cell 22, 3921–3934. 10.1105/tpc.110.074518 21183706 PMC3027163

[B33] Givnish T. J. Zuluaga A. Marques I. Lam V. K. Y. Gomez M. S. Iles W. J. D. (2016). Phylogenomics and historical biogeography of the monocot order Liliales: out of Australia and through Antarctica. Cladistics 32, 581–605. 10.1111/cla.12153 34727673

[B34] Gong Y.-B. Huang S.-Q. (2009). Floral symmetry: pollinator-mediated stabilizing selection on flower size in bilateral species. Proc. R. Soc. B Biol. Sci. 276, 4013–4020. 10.1098/rspb.2009.1254 19710062 PMC2825790

[B35] Guo Z. Fujioka S. Blancaflor E. B. Miao S. Gou X. Li J. (2010). TCP1 modulates brassinosteroid biosynthesis by regulating the expression of the key biosynthetic gene DWARF4 in *Arabidopsis thaliana*. Plant Cell 22, 1161–1173. 10.1105/tpc.109.069203 20435901 PMC2879762

[B36] Heery D. M. Kalkhoven E. Hoare S. Parker M. G. (1997). A signature motif in transcriptional co-activators mediates binding to nuclear receptors. Nature 387, 733–736. 10.1038/42750 9192902

[B37] Hileman L. C. (2014). Trends in flower symmetry evolution revealed through phylogenetic and developmental genetic advances. Philos. Trans. R. Soc. Lond. B. Biol. Sci. 369, 1–10. 10.1098/rstb.2013.0348 24958922 PMC4071522

[B38] Hileman L. C. Baum D. A. (2003). Why do paralogs persist? Molecular evolution of *CYCLOIDEA* and related floral symmetry genes in Antirrhineae (Veronicaceae). Mol. Biol. Evol. 20, 591–600. 10.1093/molbev/msg063 12679544

[B39] Horn S. Pabón-Mora N. Theuß V. S. Busch A. Zachgo S. (2015). Analysis of the CYC/TB1 class of TCP transcription factors in basal angiosperms and magnoliids. Plant J. 81, 559–571. 10.1111/tpj.12750 25557238

[B40] Hoshino Y. Igarashi T. Ohshima M. Shinoda K. Murata N. Kanno A. (2014). Characterization of *CYCLOIDEA-like* genes in controlling floral zygomorphy in the monocotyledon *Alstroemeria*. Sci. Hortic. (Amsterdam). 169, 6–13. 10.1016/j.scienta.2014.01.046

[B41] Howarth D. G. Donoghue M. J. (2006). Phylogenetic analysis of the “ECE” (CYC/TB1) clade reveals duplications predating the core eudicots. Proc. Natl. Acad. Sci. U.S.A. 103, 9101–9106. 10.1073/pnas.0602827103 16754863 PMC1482573

[B42] Howarth D. G. Martins T. Chimney E. Donoghue M. J. (2011). Diversification of *CYCLOIDEA* expression in the evolution of bilateral flower symmetry in Caprifoliaceae and *Lonicera* (Dipsacales). Ann. Bot. 107, 1521–1532. 10.1093/aob/mcr049 21478175 PMC3108805

[B43] Katoh K. Misawa K. Kuma K. Miyata T. (2002). MAFFT: a novel method for rapid multiple sequence alignment based on fast Fourier transform. Nucleic Acids Res. 30, 3059–3066. 10.1093/nar/gkf436 12136088 PMC135756

[B44] Kieffer M. Master V. Waites R. Davies B. (2011). TCP14 and TCP15 affect internode length and leaf shape in *Arabidopsis*. Plant J. 68, 147–158. 10.1111/j.1365-313X.2011.04674.x 21668538 PMC3229714

[B45] Kocyan A. (2007). The discovery of polyandry in *Curculigo* (Hypoxidaceae): implications for androecium evolution of asparagoid monocotyledons. Ann. Bot. 100, 241–248. 10.1093/aob/mcm091 17565969 PMC2735314

[B46] Kosugi S. Ohashi Y. (1997). PCF1 and PCF2 specifically bind to cis elements in the rice proliferating cell nuclear antigen gene. Plant Cell 9, 1607–1619. 10.1105/tpc.9.9.1607 9338963 PMC157037

[B47] Kosugi S. Ohashi Y. (2002). DNA binding and dimerization specificity and potential targets for the TCP protein family. Plant J. 30, 337–348. 10.1046/j.1365-313X.2002.01294.x 12000681

[B48] Koyama T. Furutani M. Tasaka M. Ohme-Takagi M. (2007). TCP transcription factors control the morphology of shoot lateral organs via negative regulation of the expression of boundary-specific genes in *Arabidopsis*. Plant Cell 19, 473–484. 10.1105/tpc.106.044792 17307931 PMC1867346

[B49] Koyama T. Mitsuda N. Seki M. Shinozaki K. Ohme-Takagi M. (2010a). TCP transcription factors regulate the activities of ASYMMETRIC LEAVES1 and miR164, as well as the auxin response, during differentiation of leaves in *Arabidopsis*. Plant Cell 22, 3574–3588. 10.1105/tpc.110.075598 21119060 PMC3015130

[B50] Koyama T. Sato F. Ohme-Takagi M. (2010b). A role of TCP1 in the longitudinal elongation of leaves in *Arabidopsis*. Biosci. Biotechnol. Biochem. 74, 2145–2147. 10.1271/bbb.100442 20944404

[B51] Li S. Lauri A. Ziemann M. Busch A. Bhave M. Zachgo S. (2009). Nuclear activity of ROXY1, a glutaredoxin interacting with TGA factors, is required for petal development in *Arabidopsis thaliana*. Plant Cell 21, 429–441. 10.1105/tpc.108.064477 19218396 PMC2660636

[B52] Lin Y. F. Chen Y. Y. Hsiao Y. Y. Shen C. Y. Hsu J. L. Yeh C. M.. (2016). Genome-wide identification and characterization of TCP genes involved in ovule development of *Phalaenopsis equestris*. J. Exp. Bot. 67, 5051–5066. 10.1093/jxb/erw273 27543606 PMC5014156

[B53] Luo D. Carpenter R. Copsey L. Vincent C. Clark J. Coen E. (1999). Control of organ asymmetry in flowers of Antirrhinum. Cell 99, 367–376. 10.1016/S0092-8674(00)81523-8 10571179

[B54] Luo D. Carpenter R. Vincent C. Copsey L. Coen E. (1996). Origin of floral asymmetry in *Antirrhinum*. Nature 383, 794–799. 10.1038/383794a0 8893002

[B55] Ma J. Liu F. Wang Q. Wang K. Jones D. C. Zhang B. (2016). Comprehensive analysis of TCP transcription factors and their expression during cotton (Gossypium arboreum) fiber early development. Sci. Rep. 6:21535. 10.1038/srep21535 26857372 PMC4746668

[B56] Martín-Trillo M. Cubas P. (2010). TCP genes: a family snapshot ten years later. Trends Plant Sci. 15, 31–39. 10.1016/j.tplants.2009.11.003 19963426

[B57] Miller M. A. Pfeiffer W. Schwartz T. (2010). Creating the CIPRES science gateway for inference of large phylogenetic trees, in Gateway Computing Environments Workshop, GCE (San Diego, CA).

[B58] Mondragón-Palomino M. (2013). Perspectives on MADS-box expression during orchid flower evolution and development. Front. Plant Sci. 4:377. 10.3389/fpls.2013.00377 24065980 PMC3779858

[B59] Mondragón-Palomino M. Theißen G. (2009). Why are orchid flowers so diverse? Reduction of evolutionary constraints by paralogues of class B floral homeotic genes. Ann. Bot. 104, 583–594. 10.1093/aob/mcn258 19141602 PMC2720651

[B60] Mondragón-Palomino M. Trontin C. (2011). High time for a roll call: gene duplication and phylogenetic relationships of TCP-like genes in monocots. Ann. Bot. 107, 1533–1544. 10.1093/aob/mcr059 21444336 PMC3108806

[B61] Nag A. King S. Jack T. (2009). miR319a targeting of TCP4 is critical for petal growth and development in *Arabidopsis*. Proc. Natl. Acad. Sci. U.S.A. 106, 22534–22539. 10.1073/pnas.0908718106 20007771 PMC2799693

[B62] Nath U. Crawford B. C. W. Carpenter R. Coen E. (2003). Genetic control of surface curvature. Am. Assoc. Adv. Sci. 299, 1404–1407. 10.1126/science.1079354 12610308

[B63] Navaud O. Dabos P. Carnus E. Tremousaygue D. Hervé C. (2007). TCP transcription factors predate the emergence of land plants. J. Mol. Evol. 65, 23–33. 10.1007/s00239-006-0174-z 17568984

[B64] Ori N. Cohen A. R. Etzioni A. Brand A. Yanai O. Shleizer S.. (2007). Regulation of LANCEOLATE by miR319 is required for compound-leaf development in tomato. Nat. Genet. 39, 787–791. 10.1038/ng2036 17486095

[B65] Pabón-Mora N. González F. (2008). Floral ontogeny of *Telipogon* spp. (Orchidaceae) and insights on the perianth symmetry in the family. Int. J. Plant Sci. 169, 1159–1173. 10.1086/591982

[B66] Palatnik J. F. Allen E. Wu X. Schommer C. Schwab R. Carrington J. C.. (2003). Control of leaf morphogenesis by microRNAs. Nature 425, 257–263. 10.1038/nature01958 12931144

[B67] Parapunova V. Busscher M. Busscher-Lange J. Lammers M. Karlova R. Bovy A. G.. (2014). Identification, cloning and characterization of the tomato TCP transcription factor family. BMC Plant Biol. 14:157. 10.1186/1471-2229-14-157 24903607 PMC4070083

[B68] Posada D. Crandall K. A. (1998). MODELTEST: testing the model of DNA substitution. Bioinformatics 14, 817–818. 10.1093/bioinformatics/14.9.817 9918953

[B69] Preston J. C. Hileman L. C. (2009). Developmental genetics of floral symmetry evolution. Trends Plant Sci. 14, 147–154. 10.1016/j.tplants.2008.12.005 19231272

[B70] Preston J. C. Hileman L. C. (2012). Parallel evolution of TCP and B-class genes in Commelinaceae flower bilateral symmetry. Evodevo 3:6. 10.1186/2041-9139-3-6 22394484 PMC3359255

[B71] Preston J. C. Kost M. A.„ Hileman L. C. (2009). Conservation and diversification of the symmetry developmental program among close relatives of snapdragon with divergent floral morphologies. New Phytol. 182, 751–762. 10.1111/j.1469-8137.2009.02794.x 19291006

[B72] Pruneda-Paz J. L. Breton G. Para A. Kay S. A. (2009). A functional genomics approach reveals CHE as a component of the Arabidopsis circadian clock. Science 323, 1481–1485. 10.1126/science.1167206 19286557 PMC4259050

[B73] Raimundo J. Sobral R. Bailey P. Azevedo H. Galego L. Almeida J.. (2013). A subcellular tug of war involving three MYB-like proteins underlies a molecular antagonism in *Antirrhinum* flower asymmetry. Plant J. 75, 527–538. 10.1111/tpj.12225 23638688

[B74] Rambaut A. (2014). FigTree: Tree Figure Drawing Tool.

[B75] Reeves P. A Olmstead R. G. (2003). Evolution of the TCP gene family in asteridae: cladistic and network approaches to understanding regulatory gene family diversification and its impact on morphological evolution. Mol. Biol. Evol. 20, 1997–2009. 10.1093/molbev/msg211 12885953

[B76] Resentini F. Felipo-Benavent A. Colombo L. Blázquez M. A. Alabadí D. Masiero S. (2015). TCP14 and TCP15 mediate the promotion of seed germination by gibberellins in *Arabidopsis thaliana*. Mol. Plant 8, 482–485. 10.1016/j.molp.2014.11.018 25655823

[B77] Rudall P. J. (2002). Unique floral structures and iterative evolutionary themes in asparagales: insights from a morphological cladistic analysis. Bot. Rev. 68, 488–509. 10.1663/0006-8101(2002)068[0488:UFSAIE

[B78] Rudall P. J. Bateman R. M. (2002). Roles of synorganisation, zygomorphy and heterotopy in floral evolution: the gynostemium and labellum of orchids and other lilioid monocots. Biol. Rev. Camb. Philos. Soc. 77, 403–441. 10.1017/S1464793102005936 12227521

[B79] Rudall P. J. Bateman R. M. (2004). Evolution of zygomorphy in monocot flowers: iterative patterns and developmental constraints. New Phytol. 162, 25–44. 10.1111/j.1469-8137.2004.01032.x

[B80] Rudall P. J. Perl C. D. Bateman R. M. (2013). Organ homologies in orchid flowers re-interpreted using the Musk Orchid as a model. PeerJ 1, 1–23. 10.7717/peerj.26 23638361 PMC3628842

[B81] Sarvepalli K. Nath U. (2011). Hyper-activation of the TCP4 transcription factor in *Arabidopsis thaliana* accelerates multiple aspects of plant maturation. Plant J. 67, 595–607. 10.1111/j.1365-313X.2011.04616.x 21518050

[B82] Schommer C. Bresso E. G. Spinelli S. V. Palatnik J. F. (2012). MicroRNAs in plant development and stress responses. Screen 15, 29–47. 10.1007/978-3-642-27384-1

[B83] Schommer C. Debernardi J. M. Bresso E. G. Rodriguez R. E. Palatnik J. F. (2014). Repression of cell proliferation by miR319-regulated TCP4. Mol. Plant 7, 1533–1544. 10.1093/mp/ssu084 25053833

[B84] Schommer C. Palatnik J. F. Aggarwal P. Chételat A. Cubas P. Farmer E. E.. (2008). Control of jasmonate biosynthesis and senescence by miR319 targets. PLoS Biol. 6:e230. 10.1371/journal.pbio.0060230 18816164 PMC2553836

[B85] Shi P. Guy K. M. Wu W. Fang B. Yang J. Zhang M.. (2016). Genome-wide identification and expression analysis of the *ClTCP* transcription factors in *Citrullus lanatus*. BMC Plant Biol. 16:85. 10.1186/s12870-016-0765-9 27072931 PMC4830022

[B86] Simpson M. G. (2006). Plant Systematics. San Diego, CA: Elsevier Academic Press.

[B87] Su C. L. Chen W. C. Lee A. Y. Chen C. Y. Chang Y. C. A. Chao Y. T.. (2013). A modified ABCDE model of flowering in orchids based on gene expression profiling studies of the moth orchid *Phalaenopsis aphrodite*. PLoS ONE 8:e80462. 10.1371/journal.pone.0080462 24265826 PMC3827201

[B88] Tähtiharju S. Rijpkema A. S. Vetterli A. Albert V. A. Teeri T. H. Elomaa P. (2012). Evolution and diversification of the *CYC/TB1* gene family in asteraceae-a comparative study in Gerbera (mutisieae) and sunflower (heliantheae). Mol. Biol. Evol. 29, 1155–1166. 10.1093/molbev/msr283 22101417

[B89] Tsai W. C. Fu C. H. Hsiao Y. Y. Huang Y. M. Chen L. J. Wang M.. (2013). OrchidBase 2.0: comprehensive collection of Orchidaceae floral transcriptomes. Plant Cell Physiol. 54, 1–8. 10.1093/pcp/pcs187 23314755

[B90] Uberti-Manassero N. G. Lucero L. E. Viola I. L. Vegetti A. C. Gonzalez D. H. (2012). The class i protein AtTCP15 modulates plant development through a pathway that overlaps with the one affected by CIN-like TCP proteins. J. Exp. Bot. 63, 809–823. 10.1093/jxb/err305 22016421

[B91] Vieira C. P. Vieira J. Charlesworth D. (1999). Evolution of the cycloidea gene family in Antirrhinum and Misopates. Mol. Biol. Evol. 16, 1474–1483. 10.1093/oxfordjournals.molbev.a026059 10555278

[B92] Wang J. Wang Y. Luo D. (2010). LjCYC genes constitute floral dorsoventral asymmetry in *Lotus japonicus*. J. Integr. Plant Biol. 52, 959–970. 10.1111/j.1744-7909.2010.00926.x 20977654

[B93] Wilfinger W. W. Mackey K. Chomczynski P. (1997). NanoDrop and design are registered trademarks of NanoDrop Technologies 260/280 and 260/230 Ratios NanoDrop® ND-1000 and ND-8000 8-Sample Spectrophotometers. Biotechniques 22, 474–481.9067025

[B94] Yang C. Li D. Mao D. Liu X. Ji C. Li X.. (2013). Overexpression of microRNA319 impacts leaf morphogenesis and leads to enhanced cold tolerance in rice (*Oryza sativa*L.). Plant Cell Environ. 36, 2207–2218. 10.1111/pce.12130 23651319

[B95] Yang X. Pang H. B. Liu B. L. Qiu Z. J. Gao Q. Wei L.. (2012). Evolution of double positive autoregulatory feedback loops in *CYCLOIDEA2* clade genes is associated with the origin of floral zygomorphy. Plant Cell 24, 1834–1847. 10.1105/tpc.112.099457 22649271 PMC3442572

[B96] Yao X. Ma H. Wang J. Zhang D. (2007). Genome-wide comparative analysis and expression pattern of TCP gene families in *Arabidopsis thaliana* and *Oryza sativa*. J. Integr. Plant Biol. 49, 885–897. 10.1111/j.1744-7909.2007.00509.x

[B97] Yuan Z. Gao S. Xue D.-W. Luo D. Li L.-T. Ding S.-Y.. (2009). RETARDED PALEA1 controls palea development and floral zygomorphy in rice. Plant Physiol. 149, 235–244. 10.1104/pp.108.128231 18952859 PMC2613737

[B98] Zhang W. Kramer E. M. Davis C. C. (2010). Floral symmetry genes and the origin and maintenance of zygomorphy in a plant-pollinator mutualism. Proc. Natl. Acad. Sci. U.S.A. 107, 6388–6393. 10.1073/pnas.0910155107 20363959 PMC2851953

[B99] Zhang W. Steinmann V. W. Nikolov L. Kramer E. M. Davis C. C. (2013). Divergent genetic mechanisms underlie reversals to radial floral symmetry from diverse zygomorphic flowered ancestors. Front. Plant Sci. 4:302. 10.3389/fpls.2013.00302 23970887 PMC3747361

[B100] Zhou M. Li D. Li Z. Hu Q. Yang C. Zhu L.. (2013). Constitutive expression of a miR319 gene alters plant development and enhances salt and drought tolerance in transgenic creeping bentgrass. Plant Physiol. 161, 1375–1391. 10.1104/pp.112.208702 23292790 PMC3585603

