# Supplementary figures and images for: Evolution and Expression Patterns of TCP Genes in Asparagales
Source: Front Plant Sci. 2017 Jan 17;8:9. doi: 10.3389/fpls.2017.00009 (PMC5239819; doi:10.3389/fpls.2017.00009)

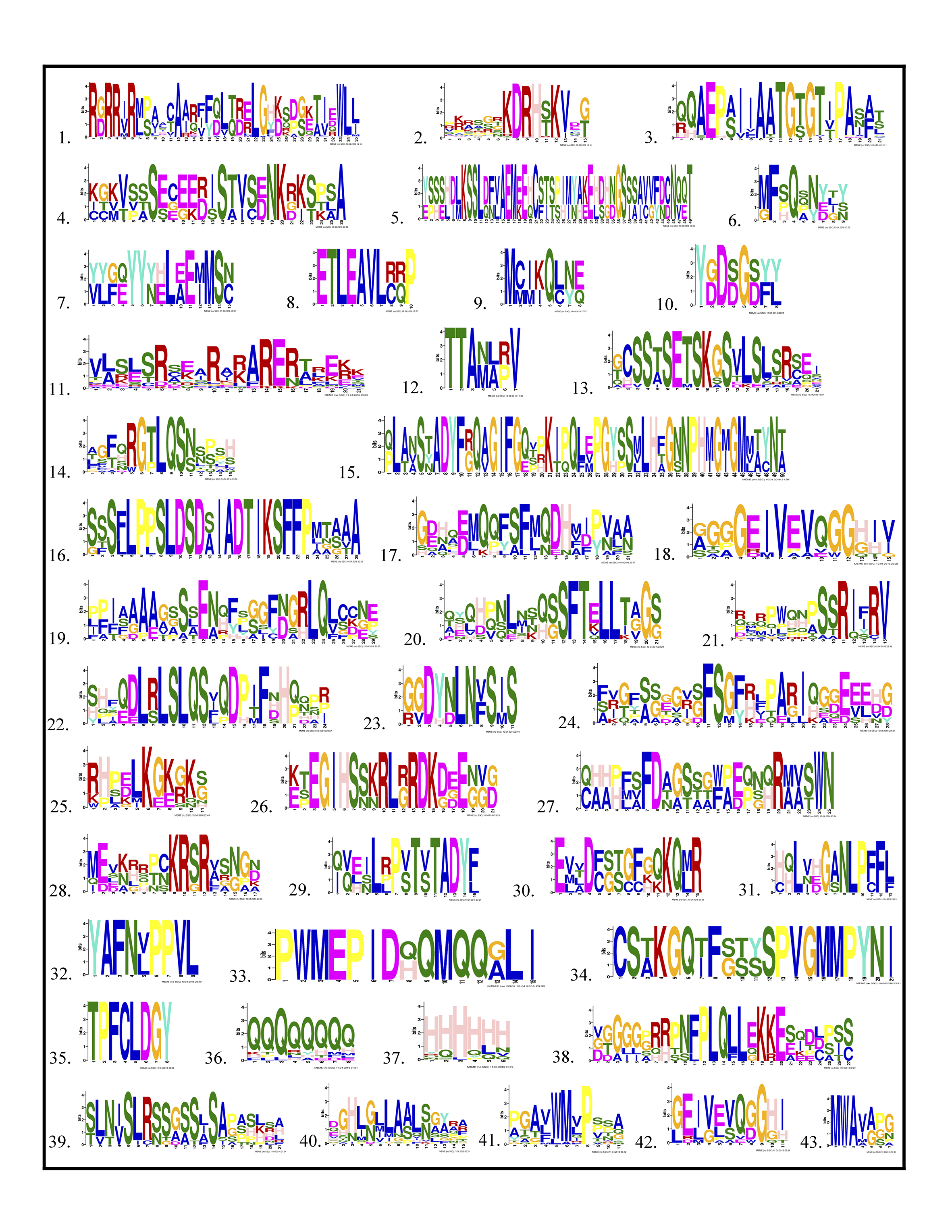

Supplement: Supplementary Figure 1 — Conserved motifs in and Orchidaceae and non-Orchidaceae Asparagales TCP-like proteins. Model core eudicots and monocots used as reference include Arabidopsis thaliana, Antirrhinum majus, and Oryza sativa. Motifs 1, 2, and 3 correspond to the conserved TCP domain. Motif 11 indicates the characteristic R domain in Class II TCP-like genes. Motif 14 corresponds to the miR319 binding site in CIN-like genes. [file Image1.TIF]

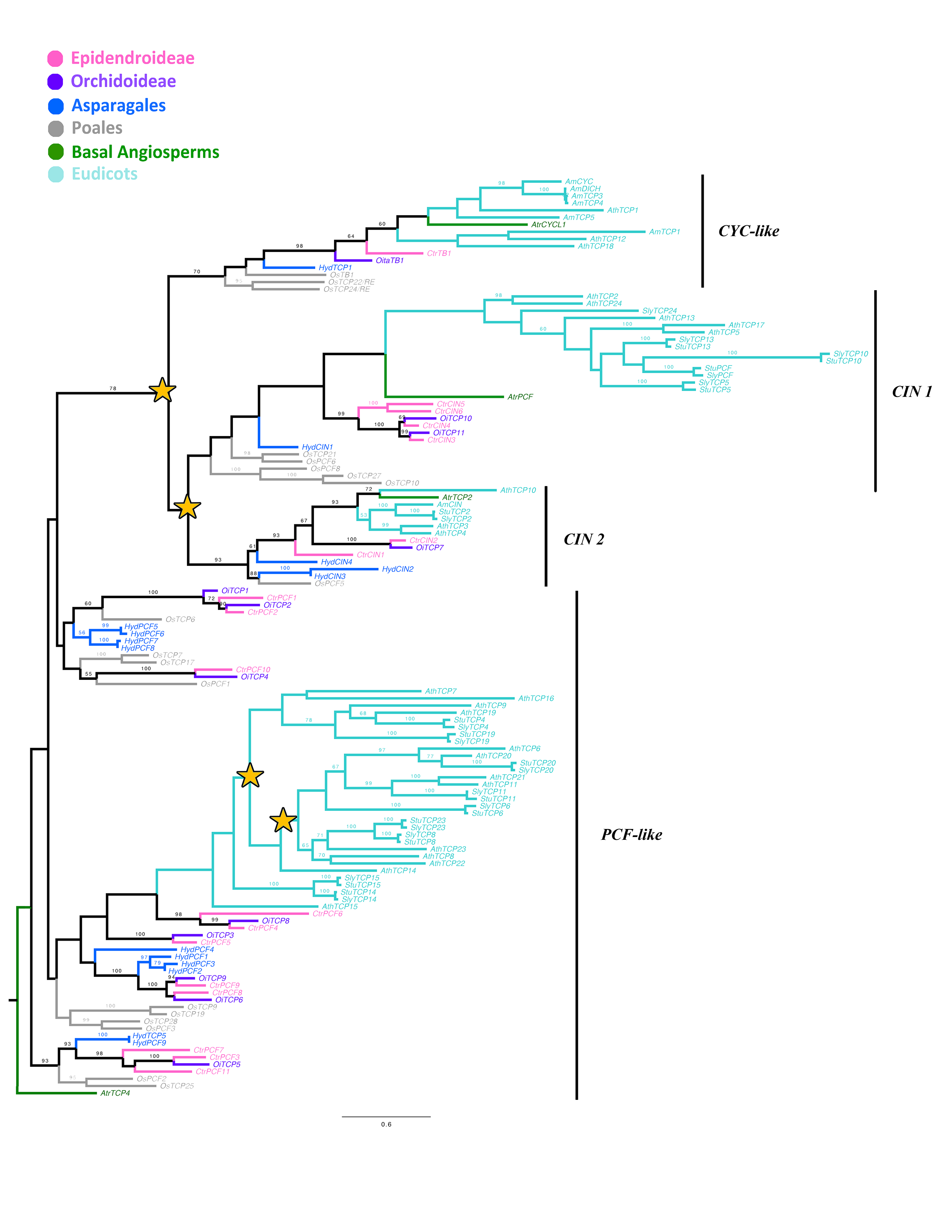

Supplement: Supplementary Figure 2 — ML analysis of TCP-like genes with extended Solanaceae sampling. ML phylogenetic analysis of TCP-like genes with reduced sampling including only model organisms like Arabidopsis thaliana, Oryza sativa, Solanum lycopersicum, Solanum tuberosum, two Orchidaceae species, Cattleya trianae, Orchis italica, one non-Orchidaceae Asparagales, Hypoxis decumbens and the early diverging angiosperm Amborella trichopoda. Yellow stars indicate large scale duplication events. TCP major clades are labeled to the right. Branch and taxa colors correspond to those in the conventions. BS values ≥ 50 are shown. [file Image2.TIF]

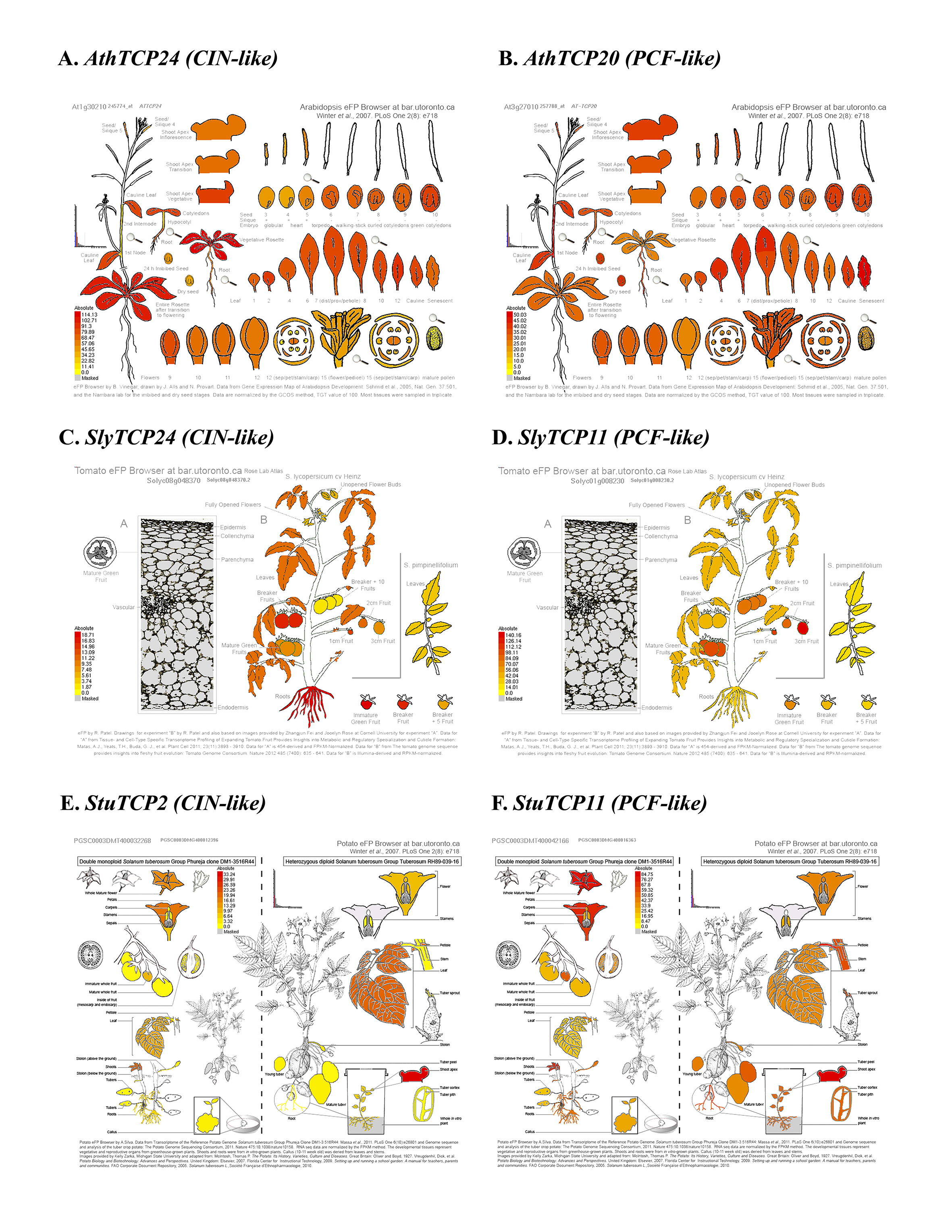

Supplement: Supplementary Figure 3 — Expression patterns of TCP-like homologs in Arabidopsis thaliana (Brassicaceae), Solanum lycopersicum and Solanum tuberosum (Solanaceae). (A) Arabidopsis thaliana AthTCP24 (CIN-like). (B) Arabidopsis thaliana AthTCP20 (PCF-like). (C) Solanum lycopersicum SlyTCP24 (CIN-like). (D) Solanum lycopersicum SlyTCP11 (PCF-like). (E) Solanum tuberosum StuTCP2 (CIN-like). (F) Solanum tuberosum StuTCP11 (PCF-like). Expression patterns show here were selected for the genes that showed the broader, most comprehensive expression patterns after comparing expression for all paralogs, and are used to summarize putative expression patterns for each gene clade. Expression levels vary in intensity between different copies of CIN-like and PCF-like. Taken from the eFP browser, last accessed Sep 5/2016 (http://bar.utoronto.ca/efp_tomato/cgi-bin/efpWeb.cgi and http://bar.utoronto.ca/efp_potato/cgi-bin/efpWeb.cgi). [file Image3.TIF]
