# Supplementary material for: Evolution and Expression Patterns of TCP Genes in Asparagales
Source: Front Plant Sci. 2017 Jan 17;8:9. doi: 10.3389/fpls.2017.00009 (PMC5239819; doi:10.3389/fpls.2017.00009)
Supplement: Supplementary Table 1 — List of sequences used in this study. [file Table1.DOCX]

**Supplementary table 1.** List of sequences used in this study.

| **GENE CODE** | **LOCUS NAME** | **DATA BASE** | **SPECIES** | **FAMILY** | **ORDEN** | **CLADE** |
| --- | --- | --- | --- | --- | --- | --- |
| *AdTCP3* | FGRF-2014698 | 1KP | *Asparagus densiflorus* | Asparagaceae | Asparagales | CIN |
| *AdTCP4* | FGRF-2016067 | 1KP | *Asparagus densiflorus* | Asparagaceae | Asparagales | CIN |
| *AlsTCP1* | GJPF-2064255 | 1KP | *Allium sativum* | Amaryllidaceae | Asparagales | CIN |
| *AlyrCIN* | 929286 | Phytozome | *Arabidopsis lyrata* | Brassicaceae | Brassicales | CIN |
| *AmCIN* | AY205603.1 | NCBI | *Antirrihnum majus* | Plantaginaceae | Lamiales | CIN |
| *ApTCP06749* | unigene6109_Ap_fb/148216 | Orchidbase | *Apostasia schenzhenica* | Orchidaceae | Asparagales | CIN |
| *AsTCP-1* | Unigene15551 | Orchidbase | *Apostasia schenzhenica* | Orchidaceae | Asparagales | CIN |
| *AsTCP-2* | Unigene12369 | Orchidbase | *Apostasia schenzhenica* | Orchidaceae | Asparagales | CIN |
| *AthTCP10* | At2g31070/NP_565712/NM_128662.2 | NCBI | *Arabidopsis thaliana* | Brassicaceae | Brassicales | CIN |
| *AthTCP13* | At3g02150/NP_850501/NM_180170.2 | NCBI | *Arabidopsis thaliana* | Brassicaceae | Brassicales | CIN |
| *AthTCP17* | At5g08070/NP_196424/NM_120889.1 | NCBI | *Arabidopsis thaliana* | Brassicaceae | Brassicales | CIN |
| *AthTCP2* | At4g18390/NP_567553/NM_117950.4 | NCBI | *Arabidopsis thaliana* | Brassicaceae | Brassicales | CIN |
| *AthTCP24* | At1g30210/NP_849730/NM_179399.2 | NCBI | *Arabidopsis thaliana* | Brassicaceae | Brassicales | CIN |
| *AthTCP3* | At1g53230/NP_564624/NM_104201.3 | NCBI | *Arabidopsis thaliana* | Brassicaceae | Brassicales | CIN |
| *AthTCP4* | At3g15030/NP_850589/NM_180258.3 | NCBI | *Arabidopsis thaliana* | Brassicaceae | Brassicales | CIN |
| *AthTCP5* | At5g60970/NP_200905/NM_125490.2 | NCBI | *Arabidopsis thaliana* | Brassicaceae | Brassicales | CIN |
| *AtrPCF* | AmTr_v1.0_scaffold00092 | www.amborella.org | *Amborella trichopoda* | Amborellaceae | Amborellales | CIN |
| *AtrTCP2* | AmTr_v1.0_scaffold00021 | www.amborella.org | *Amborella trichopoda* | Amborellaceae | Amborellales | CIN |
| *AtTCP-3* | KXSK-2003499 | 1KP | *Agave tequilana* | Asparagaceae | Asparagales | CIN |
| *AtTCP7* | KXSK-2027361 | 1KP | *Agave tequilana* | Asparagaceae | Asparagales | CIN |
| *AvTCP-1* | JVBR-2008525 | 1KP | *Aloe vera* | Xanthorrhoeaceae | Asparagales | CIN |
| *AvTCP-2* | JVBR-2011221 | 1KP | *Aloe vera* | Xanthorrhoeaceae | Asparagales | CIN |
| *AvTCP-3* | JVBR-2003769 | 1KP | *Aloe vera* | Xanthorrhoeaceae | Asparagales | CIN |
| *BosTCP-1* | EMJJ-2022730 | 1KP | *Borya sphaerocephala* | Boryaceae | Asparagales | CIN |
| *BosTCP2* | EMJJ-2020269 | 1KP | *Borya sphaerocephala* | Boryaceae | Asparagales | CIN |
| *BosTCP4* | EMJJ-2014919 | 1KP | *Borya sphaerocephala* | Boryaceae | Asparagales | CIN |
| *BsTCP-4* | IXEM-2002156 | 1KP | *Brodiaea sierrae* | Asparagaceae | Asparagales | CIN |
| *BsTCP5* | IXEM-2019613 | 1KP | *Brodiaea sierrae* | Asparagaceae | Asparagales | CIN |
| *CaruTB1* | Carubv10015851 |  | *Capsella rubella* | Brassicaceae | Brassicales | CIN |
| *CoTCP-3* | KYNE-2012588 | 1KP | *Cyanella orchidofromis* | Tecophilaeaceae | Asparagales | CIN |
| *CoTCP-4* | KYNE-2012587 | 1KP | *Cyanella orchidofromis* | Tecophilaeaceae | Asparagales | CIN |
| *CtrCIN1* | c10815_g1_i1 | UdeA | *Cattleya trianae* | Orchidaceae | Asparagales | CIN |
| *CtrCIN2* | c20571_g1_i1 | UdeA | *Cattleya trianae* | Orchidaceae | Asparagales | CIN |
| *CtrCIN3* | c20515_g2_i1 | UdeA | *Cattleya trianae* | Orchidaceae | Asparagales | CIN |
| *CtrCIN4* | c20515_g1_i1 | UdeA | *Cattleya trianae* | Orchidaceae | Asparagales | CIN |
| *CtrCIN5* | c19791_g1_i1 | UdeA | *Cattleya trianae* | Orchidaceae | Asparagales | CIN |
| *CtrCIN6* | c18629_g1_i1 | UdeA | *Cattleya trianae* | Orchidaceae | Asparagales | CIN |
| *CurTCP-2* | YJUG-2141480 | 1KP | *Curculigo sp.* | Hypoxidaceae | Asparagales | CIN |
| *CurTCP3* | YJUG-2014318 | 1KP | *Curculigo sp.* | Hypoxidaceae | Asparagales | CIN |
| *CurTCP5* | YJUG-2015545 | 1KP | *Curculigo sp.* | Hypoxidaceae | Asparagales | CIN |
| *CurTCP7* | YJUG-2018466 | 1KP | *Curculigo sp.* | Hypoxidaceae | Asparagales | CIN |
| *CycTCP5* | RDYY-2012033 | 1KP | *Cyanastrum cordifolium* | Tecophilaeaceae | Asparagales | CIN |
| *CycTCP6* | RDYY-2007044 | 1KP | *Cyanastrum cordifolium* | Tecophilaeaceae | Asparagales | CIN |
| *CyfaCIN* | KJ956809.1 | NCBI | *Cymbidium faberii* | Orchidaceae | Asparagales | CIN |
| *CyoTCP-1* | Unigene108152 | Orchidbase | *Cymbium sinense* | Orchidaceae | Asparagales | CIN |
| *CyoTCP-2* | Unigene101430 | Orchidbase | *Cymbium sinense* | Orchidaceae | Asparagales | CIN |
| *CysTCP-1* | Unigene132882 | Orchidbase | *Cypripedium singchii* | Orchidaceae | Asparagales | CIN |
| *DaTCP-1* | SVTS-2018086 | 1KP | *Drimia altissima* | Asparagaceae | Asparagales | CIN |
| *DenhTCP-1* | ABF618891/DQ517495.1 | NCBI | *Dendrobium hybrid cultivar* | Orchidaceae | Asparagales | CIN |
| *DeTCP-3* | XZME-2020610 | 1KP | *Drakea elastica* | Orchidaceae | Asparagales | CIN |
| *DeTCP4* | XZME-2016102 | 1KP | *Drakea elastica* | Orchidaceae | Asparagales | CIN |
| *DeTCP7* | XZME-2014319 | 1KP | *Drakea elastica* | Orchidaceae | Asparagales | CIN |
| *DpTCP-3* | UZXL-2002246 | 1KP | *Disporopsis pernyi* | Asparagaceae | Asparagales | CIN |
| *DpTCP-4* | UZXL-2002245 | 1KP | *Disporopsis pernyi* | Asparagaceae | Asparagales | CIN |
| *EpTCP-1b* | EPTC014051 | Orchidstra | *Erycina pusilla* | Orchidaceae | Asparagales | CIN |
| *EpTCP-2b* | EPTC005492 | Orchidstra | *Onc. Gower ramsey* | Orchidaceae | Asparagales | CIN |
| *GfTCP-1* | Unigene27438 | Orchidbase | *Galeola faberi* | Orchidaceae | Asparagales | CIN |
| *GfTCP-5* | Unigene123331 | Orchidbase | *Galeola faberi* | Orchidaceae | Asparagales | CIN |
| *GpTCP-3* | VTUS-2005814 | 1KP | *Goodyera pubescens* | Orchidaceae | Asparagales | CIN |
| *GpTCP4* | VTUS-2049649 | 1KP | *Goodyera pubescens* | Orchidaceae | Asparagales | CIN |
| *GpTCP5* | VTUS-2003026 | 1KP | *Goodyera pubescens* | Orchidaceae | Asparagales | CIN |
| *GpTCP7* | VTUS-2008003 | 1KP | *Goodyera pubescens* | Orchidaceae | Asparagales | CIN |
| *GpTCP9* | VTUS-2010182 | 1KP | *Goodyera pubescens* | Orchidaceae | Asparagales | CIN |
| *HadTCP-2* | Unigene86690 | Orchidbase | *Habenaria delavayi* | Orchidaceae | Asparagales | CIN |
| *HadTCP-3* | Unigene96363 | Orchidbase | *Habenaria delavayi* | Orchidaceae | Asparagales | CIN |
| *HdTCP-3* | LELS-2021059 | 1KP | *Haemaria discolor* | Orchidaceae | Asparagales | CIN |
| *HdTCP4* | LELS-2016290 | 1KP | *Haemaria discolor* | Orchidaceae | Asparagales | CIN |
| *HdTCP6* | LELS-2009606 | 1KP | *Haemaria discolor* | Orchidaceae | Asparagales | CIN |
| *HdTCP8* | LELS-2009440 | 1KP | *Haemaria discolor* | Orchidaceae | Asparagales | CIN |
| *HdTCP9* | LELS-2009441 | 1KP | *Haemaria discolor* | Orchidaceae | Asparagales | CIN |
| *HemTCP-3* | JHUL-2024050 | 1KP | *Hemerocallis sp.* | Xanthorrhoeaceae | Asparagales | CIN |
| *HfTCP-1* | Unigene104672 | Orchidbase | *Hemipilia forrestii* | Orchidaceae | Asparagales | CIN |
| *HfTCP-2* | Unigene93361 | Orchidbase | *Hemipilia forrestii* | Orchidaceae | Asparagales | CIN |
| *HfTCP-3* | Unigene113091 | Orchidbase | *Hemipilia forrestii* | Orchidaceae | Asparagales | CIN |
| *HpTCP6* | CMCY-2011646 | 1KP | *Hesperaloe parviflora* | Asparagaceae | Asparagales | CIN |
| *HpTCP7* | CMCY-2011647 | 1KP | *Hesperaloe parviflora* | Asparagaceae | Asparagales | CIN |
| *HydCIN1* | c26829_g2_i1 | UdeA | *Hypoxis decumbens* | Hypoxidaceae | Asparagales | CIN |
| *HydCIN2* | c25446_g2_i3 | UdeA | *Hypoxis decumbens* | Hypoxidaceae | Asparagales | CIN |
| *HydCIN3* | c25446_g2_i4 | UdeA | *Hypoxis decumbens* | Hypoxidaceae | Asparagales | CIN |
| *HydCIN4* | c25446_g2_i2 | UdeA | *Hypoxis decumbens* | Hypoxidaceae | Asparagales | CIN |
| *JpTCP-1* | WTDE-2022300 | 1KP | *Johnsonia pubescens* | Xanthorrhoeaceae | Asparagales | CIN |
| *LlTCP-3* | MUMD-2023206 | 1KP | *Lomandra longifolia* | Asparagaceae | Asparagales | CIN |
| *McTCP-6* | XFJG-2009329 | 1KP | *Majanthenum canadense* | Asparagaceae | Asparagales | CIN |
| *McTCP7* | XFJG-2001196 | 1KP | *Maianthenum canadense* | Asparagaceae | Asparagales | CIN |
| *McTCP8* | XFJG-1001197 | 1KP | *Maianthenum canadense* | Asparagaceae | Asparagales | CIN |
| *MetrTCP18* |  |  | *Medicago truncatula* | Fabaceae | Fabales | CIN |
| *MyTCP-1* | JSAG-2003898 | 1KP | *Masdevallia yungasensis* | Orchidaceae | Asparagales | CIN |
| *MyTCP5* | JSAG-2003898 | 1KP | *Masdevallia yungasensis* | Orchidaceae | Asparagales | CIN |
| *MyTCP6* | JSAG-2010240 | 1KP | *Masdevallia yungasensis* | Orchidaceae | Asparagales | CIN |
| *NaTCP2* | HOKG-2112169 | 1KP | *Nolina atopocarpa* | Asparagaceae | Asparagales | CIN |
| *NaTCP3* | HOKG-2014584 | 1KP | *Nolina atopocarpa* | Asparagaceae | Asparagales | CIN |
| *NbTCP7* | RQZP-2024990 | 1KP | *Nolina bigelovii* | Asparagaceae | Asparagales | CIN |
| *NlTCP-3* | Unigene32573 | Orchidbase | *Neuwiedia malipoensis* | Orchidaceae | Asparagales | CIN |
| *NvTCP-4* | XEUV-2024473 | 1KP | *Narcissus viridiflorus* | Amaryllidaceae | Asparagales | CIN |
| *OiTCP10* | comp16313 | De Paolo et. al 2015 | *Orchis italica* | Orchidaceae | Asparagales | CIN |
| *OiTCP11* | comp5062 | De Paolo et. al 2015 | *Orchis italica* | Orchidaceae | Asparagales | CIN |
| *OiTCP7* | comp1326 | De Paolo et. al 2015 | *Orchis italica* | Orchidaceae | Asparagales | CIN |
| *OncTCP-1b* | OGTC014653 | Orchidstra | *Onc. Gower ramsey* | Orchidaceae | Asparagales | CIN |
| *OncTCP-2b* | OGTC013772 | Orchidstra | *Onc. Gower ramsey* | Orchidaceae | Asparagales | CIN |
| *OncTCP-3b* | OGTC012209 | Orchidstra | *Onc. Gower ramsey* | Orchidaceae | Asparagales | CIN |
| *OsPCF5* | LOC_Os01g11550 | Phytozome | *Oryza sativa* | Poaceae | Poales | CIN |
| *OsPCF6* | LOC_Os03g57190 | Phytozome | *Oryza sativa* | Poaceae | Poales | CIN |
| *OsPCF8* | LOC_Os12g42190 | Phytozome | *Oryza sativa* | Poaceae | Poales | CIN |
| *OsTCP10* | LOC_Os02g51310 | Phytozome | *Oryza sativa* | Poaceae | Poales | CIN |
| *OsTCP21* | LOC_Os07g05720 | Phytozome | *Oryza sativa* | Poaceae | Poales | CIN |
| *OsTCP27* | LOC_Os12g02090 | Phytozome | *Oryza sativa* | Poaceae | Poales | CIN |
| *PaaTCP-2* | Unigene119858 | Orchidbase | *Paphiopedilum armeniacum* | Orchidaceae | Asparagales | CIN |
| *PaaTCP-3* | Unigene106372 | Orchidbase | *Paphiopedilum armeniacum* | Orchidaceae | Asparagales | CIN |
| *PaPCF* | PATC134871 | Orchidstra | *Phalaenopsis aphrodite* | Orchidaceae | Asparagales | CIN |
| *PaTCP1b* | PATC129038 | Orchidstra | *Phalaenopsis aphrodite* | Orchidaceae | Asparagales | CIN |
| *PaTCP-1c* | PATC135086 | Orchidstra | *Phalaenopsis aphrodite* | Orchidaceae | Asparagales | CIN |
| *PaTCP-2b* | PATC133065 | Orchidstra | *Phalaenopsis aphrodite* | Orchidaceae | Asparagales | CIN |
| *PETCP06996* | PEQU_06996 | Orchidbase | *Phalaenopsis equestris* | Orchidaceae | Asparagales | CIN |
| *PETCP08153* | PEQU_08153 | Orchidbase | *Phalaenopsis equestris* | Orchidaceae | Asparagales | CIN |
| *PETCP14328* | PEQU_14328 | Orchidbase | *Phalaenopsis equestris* | Orchidaceae | Asparagales | CIN |
| *PETCP19547* | PEQU_19547 | Orchidbase | *Phalaenopsis equestris* | Orchidaceae | Asparagales | CIN |
| *PETCP21454* | PEQU_21454 | Orchidbase | *Phalaenopsis equestris* | Orchidaceae | Asparagales | CIN |
| *PeTCP-3* | Unigene62020 | Orchidbase | *Phalaenopsis equestris* | Orchidaceae | Asparagales | CIN |
| *PeTCP-4* | Unigene58430 | Orchidbase | *Phalaenopsis equestris* | Orchidaceae | Asparagales | CIN |
| *PeTCP-5* | Unigene81320 | Orchidbase | *Phalaenopsis equestris* | Orchidaceae | Asparagales | CIN |
| *PeTCP-6* | Unigene90388 | Orchidbase | *Phalaenopsis equestris* | Orchidaceae | Asparagales | CIN |
| *PeTCP-9* | Unigene82788 | Orchidbase | *Phalaenopsis equestris* | Orchidaceae | Asparagales | CIN |
| *PeTCPa* | singletons 48013/79971 | Orchidbase | *Phalaenopsis equestris* | Orchidaceae | Asparagales | CIN |
| *PeTCPc* | singletons 15918/53775 | Orchidbase | *Phalaenopsis equestris* | Orchidaceae | Asparagales | CIN |
| *PhamTB1* | unigene19087_Pa_fb | Orchidbase | *Phaphiopedilum armeniacum* | Orchidaceae | Asparagales | CIN |
| *PhamTB1b* | unigene119858_Pa_fb/514107 | Orchidbase | *Phaphiopedilum armeniacum* | Orchidaceae | Asparagales | CIN |
| *PhaTCP-1* | HQ439603.1 | NCBI | *Phalaenopsis hybrid cult.* | Orchidaceae | Asparagales | CIN |
| *PhaTCP-2* | HQ439604.1 | NCBI | *Phalaenopsis hybrid cult.* | Orchidaceae | Asparagales | CIN |
| *PhaTCP-3* | HQ439605.1 | NCBI | *Phalaenopsis hybrid cult.* | Orchidaceae | Asparagales | CIN |
| *PhaTCP-5* | HQ439607.1 | NCBI | *Phalaenopsis hybrid cult.* | Orchidaceae | Asparagales | CIN |
| *PlcTCP-1* | MTHW-2011485 | 1KP | *Platanthera clavellata* | Orchidaceae | Asparagales | CIN |
| *PtTCP-4* | FCEL-2002088 | 1KP | *Phormium tenax* | Xanthorrhoeaceae | Asparagales | CIN |
| *RpTCP-3* | JDTY-2005345 | 1KP | *Rhodophiaia pratensis* | Amaryllidaceae | Asparagales | CIN |
| *RpTCP-4* | JDTY-2005344 | 1KP | *Rhodophiaia pratensis* | Amaryllidaceae | Asparagales | CIN |
| *RpTCP-5* | JDTY-2005346 | 1KP | *Rhodophiaia pratensis* | Amaryllidaceae | Asparagales | CIN |
| *RusTCP3* | LSJW-2015984 | 1KP | *Ruscus sp.* | Asparagaceae | Asparagales | CIN |
| *SaTCP-1* | LTZF-2012628 | 1KP | *Sisyrinchium angustifolium* | Iridaceae | Asparagales | CIN |
| *StTCP-3* | MVRF-2001232 | 1KP | *Sansevieria trifasciata* | Asparagaceae | Asparagales | CIN |
| *StTCP-4* | MVRF-2001231 | 1KP | *Sansevieria trifasciata* | Asparagaceae | Asparagales | CIN |
| *StTCP8* | MVRF-2016686 | 1KP | *Sansevieria trifasciata* | Asparagaceae | Asparagales | CIN |
| *UmTCP-3* | KOFB-2002645 | 1KP | *Urginea maritima* | Asparagaceae | Asparagales | CIN |
| *VpTCP-1* | THDM-2001231 | 1KP | *Vanilla planifolia* | Orchidaceae | Asparagales | CIN |
| *VpTCP2* | THDM-2014434 | 1KP | *Vanilla planifolia* | Orchidaceae | Asparagales | CIN |
| *VpTCP4* | THDM-2002722 | 1KP | *Vanilla planifolia* | Orchidaceae | Asparagales | CIN |
| *VsTCP-1* | Unigene182983 | Orchidbase | *Vanilla shenzhenica* | Orchidaceae | Asparagales | CIN |
| *VsTCP-2* | Unigene176884 | Orchidbase | *Vanilla shenzhenica* | Orchidaceae | Asparagales | CIN |
| *VsTCP-3* | Unigene168532 | Orchidbase | *Vanilla shenzhenica* | Orchidaceae | Asparagales | CIN |
| *VsTCP-4* | Unigene123598 | Orchidbase | *Vanilla shenzhenica* | Orchidaceae | Asparagales | CIN |
| *XcTCP-6* | SART-2012619 | 1KP | *Xeronema callistemon* | Xeronemataceae | Asparagales | CIN |
| *YbTCP-3* | YBML-2025284 | 1KP | *Yucca brevifolia* | Asparagaceae | Asparagales | CIN |
| *YbTCP4* | YBML-2003766 | 1KP | *Yucca brevifolia* | Asparagaceae | Asparagales | CIN |
| *YbTCP6* | YBML-2025281 | 1KP | *Yucca brevifolia* | Asparagaceae | Asparagales | CIN |
| *YbTCP8* | YBML-2025282 | 1KP | *Yucca brevifolia* | Asparagaceae | Asparagales | CIN |
| *YfTCP6* | ICNN-2000246 | 1KP | *Yucca filamentosa* | Asparagaceae | Asparagales | CIN |
| *YfTCP7* | ICNN-2000243 | 1KP | *Yucca filamentosa* | Asparagaceae | Asparagales | CIN |
| *SlyPCF* | Solyc02g065800.1.1 | Phytozome | *Solanum lycopersicum* | Solanaceae | Solanales | CIN |
| *StuPCF* | PGSC0003DMT400068332 | Phytozome | *Solanum tuberosum* | Solanaceae | Solanales | CIN |
| *SlyTCP2* | Solyc07g062680.1.1 | Phytozome | *Solanum lycopersicum* | Solanaceae | Solanales | CIN |
| *StuTCP2* | PGSC0003DMT400032268 | Phytozome | *Solanum tuberosum* | Solanaceae | Solanales | CIN |
| *SlyTCP10* | Solyc07g053410.2.1 | Phytozome | *Solanum lycopersicum* | Solanaceae | Solanales | CIN |
| *StuTCP10* | PGSC0003DMT400024329 | Phytozome | *Solanum tuberosum* | Solanaceae | Solanales | CIN |
| *SlyTCP5* | Solyc06g069460.1.1 | Phytozome | *Solanum lycopersicum* | Solanaceae | Solanales | CIN |
| *StuTCP5* | PGSC0003DMT400010453 | Phytozome | *Solanum tuberosum* | Solanaceae | Solanales | CIN |
| *SlyTCP13* | Solyc02g089020.1.1 | Phytozome | *Solanum lycopersicum* | Solanaceae | Solanales | CIN |
| *StuTCP13* | PGSC0003DMT400003453 | Phytozome | *Solanum tuberosum* | Solanaceae | Solanales | CIN |
| *SlyTCP24* | Solyc08g048370.2.1 | Phytozome | *Solanum lycopersicum* | Solanaceae | Solanales | CIN |
| *AarCYCL1* | KP242030 | NCBI | *Aristolochia arborea* | Aristolochiaceae | Piperales | CYC/TB1 |
| *AarCYCL2* | KP242031 | NCBI | *Aristolochia arborea* | Aristolochiaceae | Piperales | CYC/TB1 |
| *AaTCP1* | AB714967.1 | NCBI | *Alstroemeria aurea* | Alstroemeriaceae | Liliales | CYC/TB1 |
| *AfCYCL1* | KP242029.1 | NCBI | *Aristolochia fimbriata* | Aristolochiaceae | Piperales | CYC/TB1 |
| *AlyrTCP1* | 338960 | Phytozome | *Arabidopsis lyrata* | Brassicaceae | Brassicales | CYC/TB1 |
| *AlyrTCP12* | 315900 | Phytozome | *Arabidopsis lyrata* | Brassicaceae | Brassicales | CYC/TB1 |
| *AlyrTCP18* | 929736 | Phytozome | *Arabidopsis lyrata* | Brassicaceae | Brassicales | CYC/TB1 |
| *AmCYC* | Y16313.1 | NCBI | *Antirrihnum majus* | Plantaginaceae | Lamiales | CYC/TB1 |
| *AmDICH* | AF199465.1 | NCBI | *Antirrihnum majus* | Plantaginaceae | Lamiales | CYC/TB1 |
| *AmTCP1* | AY168140 | NCBI | *Antirrhinum majus* | Plantaginaceae | Lamiales | CYC/TB1 |
| *AmTCP3* | AY168142.1 | NCBI | *Antirrihnum majus* | Plantaginaceae | Lamiales | CYC/TB1 |
| *AmTCP4* | AY168143.1 | NCBI | *Antirrihnum majus* | Plantaginaceae | Lamiales | CYC/TB1 |
| *AmTCP5* | AY168144 | NCBI | *Antirrhinum majus* | Plantaginaceae | Lamiales | CYC/TB1 |
| *ApTCP2* | AB714469.1 | NCBI | *Alstroemeria peregrina* | Alstroemeriaceae | Liliales | CYC/TB1 |
| *AqcTCP1* | Aquca_002_01093 | Phytozome | *Aquilegia coerulea* | Ranunculaceae | Ranunculales | CYC/TB1 |
| *AqcTCP12* | Aquca_026_00208 | Phytozome | *Aquilegia coerulea* | Ranunculaceae | Ranunculales | CYC/TB1 |
| *ArmeCyL1* | DQ347819 | NCBI | *Romneya coulteri* | Papaveraceae | Ranunculales | CYC/TB1 |
| *ArmeCyL1b* | KJ401950 | NCBI | *Romneya coulteri* | Papaveraceae | Ranunculales | CYC/TB1 |
| *AthTCP1* | NM_001160982.1 | NCBI | *Arabidopsis thaliana* | Brassicaceae | Brassicales | CYC/TB1 |
| *AthTCP12* | At1g68800/NP_177047/NM_105554.2 | NCBI | *Arabidopsis thaliana* | Brassicaceae | Brassicales | CYC/TB1 |
| *AthTCP18* | At3g18550/NP_188485/NM_112741.2 | NCBI | *Arabidopsis thaliana* | Brassicaceae | Brassicales | CYC/TB1 |
| *AtrCYCL1* | gnl\|Ambo_Trinity\|comp55302_c0_seq1 | www.amborella.org | *Amborella trichopoda* | Amborellaceae | Amborellales | CYC/TB1 |
| *BofrCyL1* |  | UDEA | *Bocconia frutescens* | Papaveraceae | Ranunculales | CYC/TB1 |
| *BradTCP1* | Bradi3g36590/XM_003572177 | Phytozome/NCBI | *Brachypodium distachyon* | Poaceae | Poales | CYC/TB1 |
| *BradTCP12/BradTB1l* | Bradi1g11060/XM_003559465 | Phytozome/NCBI | *Brachypodium distachyon* | Poaceae | Poales | CYC/TB1 |
| *BradTCP1b* | Bradi4g29980/XM_003578052 | Phytozome/NCBI | *Brachypodium distachyon* | Poaceae | Poales | CYC/TB1 |
| *BraraTCP1* | Bra004097 |  | *Brassica rapa* | Brassicaceae | Brassicales | CYC/TB1 |
| *BraraTCP12* | Bra038350 |  | *Brassica rapa* | Brassicaceae | Brassicales | CYC/TB1 |
| *BraraTCP18* | Bra001710 |  | *Brassica rapa* | Brassicaceae | Brassicales | CYC/TB1 |
| *CapaTCP1* | evm.TU.supercontig_17929 | Phytozome | *Carica papaya* | Caricaceae | Brassicales | CYC/TB1 |
| *CapaTCP12* | evm.TU.supercontig_1401.1 | Phytozome | *Carica papaya* | Caricaceae | Brassicales | CYC/TB1 |
| *CapaTCP18* | evm.TU.supercontig_13.18 | Phytozome | *Carica papaya* | Caricaceae | Brassicales | CYC/TB1 |
| *CaruTCP1* | Carubv10020555m.g. | Phytozome | *Capsella rubella* | Brassicaceae | Brassicales | CYC/TB1 |
| *CaruTCP12* | Carubv10020487m.g. | Phytozome | *Capsella rubella* | Brassicaceae | Brassicales | CYC/TB1 |
| *CaruTCP18* | Carubv10013748m.g. | Phytozome | *Capsella rubella* | Brassicaceae | Brassicales | CYC/TB1 |
| *CaseCyL1* | DQ659310 | NCBI | *Capnoides sempervirens* | Fumariaceae | Ranunculales | CYC/TB1 |
| *CaseCyL2* | DQ659311 | NCBI | *Capnoides sempervirens* | Fumariaceae | Ranunculales | CYC/TB1 |
| *CcTB1a* | JQ622131 | NCBI | *Commelina communis* | Commelinaceae | Commelinales | CYC/TB1 |
| *CcTB1b* | JQ622134 | NCBI | *Commelina communis* | Commelinaceae | Commelinales | CYC/TB1 |
| *CdTB1a* | JQ622142 | NCBI | *Commelina dianthifolia* | Commelinaceae | Commelinales | CYC/TB1 |
| *CdTB1b* | JQ622135 | NCBI | *Commelina dianthifolia* | Commelinaceae | Commelinales | CYC/TB1 |
| *ChemaCyL1* | DQ659308 | NCBI | *Chelidonium majus* | Papaveraceae | Ranunculales | CYC/TB1 |
| *ChemaCyL2* | DQ659309 | NCBI | *Chelidonium majus* | Papaveraceae | Ranunculales | CYC/TB1 |
| *CicleTCP1* | ciclev10007109m.g. | Phytozome | *Citrus clementina* | Rutaceae | Sapindales | CYC/TB1 |
| *CicleTCP12* | ciclev10033382m.g. | Phytozome | *Citrus clementina* | Rutaceae | Sapindales | CYC/TB1 |
| *CicleTCP1b* | ciclev10012612m.g. | Phytozome | *Citrus clementina* | Rutaceae | Sapindales | CYC/TB1 |
| *CirCYCL1* | HQ599293 | NCBI | *Circaeaster agrestis* | Circaeasteraceae | Ranunculales | CYC/TB1 |
| *CirCYCL1b* | HQ599294 | NCBI | *Circaeaster agrestis* | Circaeasteraceae | Ranunculales | CYC/TB1 |
| *CisiTCP1* | Orange1.1g047495 m.g. | Phytozome | *Citrus sinensis* | Rutaceae | Sapindales | CYC/TB1 |
| *CisiTCP12* | Orange1.1g039323 m.g. | Phytozome | *Citrus sinensis* | Rutaceae | Sapindales | CYC/TB1 |
| *CisiTCP18* | Orange1.1g035911 m.g. | Phytozome | *Citrus sinensis* | Rutaceae | Sapindales | CYC/TB1 |
| *CtrTB1* | c62044_g1_i1/c74262_g1_i1 | UdeA | *Cattleya trianae* | Orchidaceae | Asparagales | CYC/TB1 |
| *CurTB1* | YJUG-2138874 | 1KP | *Curculigo sp.* | Hypoxidaceae | Asparagales | CYC/TB1 |
| *CusaTCP1* | Cucsa238090 |  | *Cucumis sativus* | Cucurbitaceae | Cucurbitales | CYC/TB1 |
| *CusaTCP12* | Cucsa280880 |  | *Cucumis sativus* | Cucurbitaceae | Cucurbitales | CYC/TB1 |
| *CusaTCP1b* | Cucsa031600 |  | *Cucumis sativus* | Cucurbitaceae | Cucurbitales | CYC/TB1 |
| *CyveCyL1* | DQ659312 | NCBI | *Cysticapnos vesicarius* | Papaveraceae | Ranunculales | CYC/TB1 |
| *CyveCyL2A* | DQ659313 | NCBI | *Cysticapnos vesicarius* | Papaveraceae | Ranunculales | CYC/TB1 |
| *CyveCyL2B* | DQ659314 | NCBI | *Cysticapnos vesicarius* | Papaveraceae | Ranunculales | CYC/TB1 |
| *DitoCyL1* | DQ659315 | NCBI | *Dicentra torulosa* | Papaveraceae | Ranunculales | CYC/TB1 |
| *DitoCyL2* | DQ659316 | NCBI | *Dicentra torulosa* | Papaveraceae | Ranunculales | CYC/TB1 |
| *EugrTCP1* | EucgrG02354 |  | *Eucalyptus grandis* | Myrtaceae | Myrtales | CYC/TB1 |
| *EugrTCP18* | EucgrG02354 |  | *Eucalyptus grandis* | Myrtaceae | Myrtales | CYC/TB1 |
| *FraveTCP1* | 10333v.1.0 |  | *Fragaria vesca* | Rosaceae | Rosales | CYC/TB1 |
| *FraveTCP12* | 03882v.1.0 |  | *Fragaria vesca* | Rosaceae | Rosales | CYC/TB1 |
| *FraveTCP18* | 26211v.1.0 |  | *Fragaria vesca* | Rosaceae | Rosales | CYC/TB1 |
| *GhCYC1* | EU429302 | NCBI | *Gerbera hybrid* | Asteraceae | Asterales | CYC/TB1 |
| *GhCYC10* | JN190064 | NCBI | *Gerbera hybrida* | Asteraceae | Asterales | CYC/TB1 |
| *GhCYC2* | EU429303 | NCBI | *Gerbera hybrida* | Asteraceae | Asterales | CYC/TB1 |
| *GhCYC3* |  | NCBI | *Gerbera hybrida* | Asteraceae | Asterales | CYC/TB1 |
| *GhCYC4* |  | NCBI | *Gerbera hybrida* | Asteraceae | Asterales | CYC/TB1 |
| *GhCYC5* | JN190059 | NCBI | *Gerbera hybrida* | Asteraceae | Asterales | CYC/TB1 |
| *GhCYC6* | JN190060 | NCBI | *Gerbera hybrida* | Asteraceae | Asterales | CYC/TB1 |
| *GhCYC7* | JN190061 | NCBI | *Gerbera hybrida* | Asteraceae | Asterales | CYC/TB1 |
| *GhCYC8* | JN190062 | NCBI | *Gerbera hybrida* | Asteraceae | Asterales | CYC/TB1 |
| *GhCYC9* | JN190063 | NCBI | *Gerbera hybrida* | Asteraceae | Asterales | CYC/TB1 |
| *GlymTCP1* | Glyma08g28691 | Phytozome | *Glycine max* | Fabaceae | Fabales | CYC/TB1 |
| *GlymTCP18* | Glyma06g23410 | Phytozome | *Glycine max* | Fabaceae | Fabales | CYC/TB1 |
| *GlymTCP18b* | Glyma04g22407 | Phytozome | *Glycine max* | Fabaceae | Fabales | CYC/TB1 |
| *GlymTCP18c* | Glyma05g07943 | Phytozome | *Glycine max* | Fabaceae | Fabales | CYC/TB1 |
| *GlymTCP1b* | Glyma18g51581 | Phytozome | *Glycine max* | Fabaceae | Fabales | CYC/TB1 |
| *GlymTCP1c* | Glyma19g05921 | Phytozome | *Glycine max* | Fabaceae | Fabales | CYC/TB1 |
| *GlymTCP1d* | Glyma13g07475 | Phytozome | *Glycine max* | Fabaceae | Fabales | CYC/TB1 |
| *GoraTCP1* | Gorai.0016200400 | Phytozome | *Gossypium raimondii* | Malvaceae | Malvales | CYC/TB1 |
| *GoraTCP12* | Gorai.0086186800 | Phytozome | *Gossypium raimondii* | Malvaceae | Malvales | CYC/TB1 |
| *GoraTCP18* | Gorai.0076007500 | Phytozome | *Gossypium raimondii* | Malvaceae | Malvales | CYC/TB1 |
| *GoraTCP18b* | Gorai.0086285300 | Phytozome | *Gossypium raimondii* | Malvaceae | Malvales | CYC/TB1 |
| *GuntCYCL1* | HQ599272 | NCBI | *Gunnera tinctoria* | Gunneraceae | Gunnerales | CYC/TB1 |
| *GuntCYCL2* | HQ599273 | NCBI | *Gunnera tinctoria* | Gunneraceae | Gunnerales | CYC/TB1 |
| *HydTB1* | c52607_g1_i1 | UdeA | *Hypoxis decumbens* | Hypoxidaceae | Asparagales | CYC/TB1 |
| *HyproCyL1* | DQ659317 | NCBI | *Hypecoum procumbens* | Papaveraceae | Ranunculales | CYC/TB1 |
| *HyproCyL2* | DQ659318 | NCBI | *Hypecoum procumbens* | Papaveraceae | Ranunculales | CYC/TB1 |
| *IaTCP1* |  |  |  |  |  | CYC/TB1 |
| *LaspeCyL1* | DQ659319 | NCBI | *Lamprocapnos spectabilis* | Papaveraceae | Ranunculales | CYC/TB1 |
| *LaspeCyL2* | DQ659320 | NCBI | *Lamprocapnos spectabilis* | Papaveraceae | Ranunculales | CYC/TB1 |
| *LecoCYCL1* | HQ599287 | NCBI | *Leucospermum cordifolium* | Proteaceae | Proteales | CYC/TB1 |
| *LecoCYCL2* | HQ599288 | NCBI | *Leucospermum cordifolium* | Proteaceae | Proteales | CYC/TB1 |
| *LtCYC1* | gnl\|Liriodendron\|b4_c10975 |  | *Liriodendron tulipifera* | Magnoliaceae | Magnoliales | CYC/TB1 |
| *MemyCYCL1* | HQ599279 | NCBI | *Meliosma myriantha* | Sabiaceae | Proteales | CYC/TB1 |
| *MemyCYCL2* | HQ599280 | NCBI | *Meliosma myriantha* | Sabiaceae | Proteales | CYC/TB1 |
| *MesTCP1* |  |  |  |  |  | CYC/TB1 |
| *MesTCP12* |  |  |  |  |  | CYC/TB1 |
| *MesTCP18* |  |  |  |  |  | CYC/TB1 |
| *MetrTCP1* | AC233140_70 |  | *Medicago truncatula* | Fabaceae | Fabales | CYC/TB1 |
| *MiguTCP1* | Mgv1a025043 |  | *Mimulus guttatus* | Phrymaceae | Lamiales | CYC/TB1 |
| *MiguTCP18* | Mgv1a022535 |  | *Mimulus guttatus* | Phrymaceae | Lamiales | CYC/TB1 |
| *MiguTCP1b* | Mgv1a017886 |  | *Mimulus guttatus* | Phrymaceae | Lamiales | CYC/TB1 |
| *NaCYCL1* |  |  | *Nymphaea alba* | Nymphaeaceae | Nymphaeales | CYC/TB1 |
| *NadCYCL1* | gnl\|Nuphar\|b3_c144348 |  | *Nuphar advena* | Nymphaeaceae | Nymphaeales | CYC/TB1 |
| *NenuCYCL1* | HQ599281 | NCBI | *Nelumbo nucifera* | Nelumbonaceae | Proteales | CYC/TB1 |
| *NenuCYCL2* | HQ599282 | NCBI | *Nelumbo nucifera* | Nelumbonaceae | Proteales | CYC/TB1 |
| *NlCYCl1* | KP242027 | NCBI | *Nuphar lutea* | Nymphaeaceae | Nymphaeales | CYC/TB1 |
| *NuaCYCL1* | gnl\|Nuphar\|b3_C144348 | www.ancangio.uga.org | *Nuphar advena* | Nymphaeaceae | Nymphaeales | CYC/TB1 |
| *OitaTB1* | KR858306 | NCBI/De Paolo et. al 2015 | *Orchis italica* | Orchidaceae | Asparagales | CYC/TB1 |
| *OsTB1* | LOC_Os03g49880 | Phytozome | *Oryza sativa* | Poaceae | Poales | CYC/TB1 |
| *OsTCP22/REP1b* | LOC_Os08g33530 | Phytozome | *Oryza sativa* | Poaceae | Poales | CYC/TB1 |
| *OsTCP24/REP1* | LOC_Os09g24480 | Phytozome | *Oryza sativa* | Poaceae | Poales | CYC/TB1 |
| *ParCYCL1* | 1 gnllPersealb4_c14475 |  | *Persea americana* | Lauraceae | Laurales | CYC/TB1 |
| *ParCYCL2* | 2 gnllPersealb4_ep_c50318 |  | *Persea americana* | Lauraceae | Laurales | CYC/TB1 |
| *ParCYCL3* | 3 gnllPersealb4_c82767 |  | *Persea americana* | Lauraceae | Laurales | CYC/TB1 |
| *PaTB1* | PATC092289 | Orchidstra | *Phalaenopsis aphrodite* | Orchidaceae | Asparagales | CYC/TB1 |
| *PaTCP06749* | PATC039158 | Orchidstra | *Phalaenopsis aphrodite* | Orchidaceae | Asparagales | CYC/TB1 |
| *PETCP06749* | PEQU_06749 | Orchidbase | *Phalaenopsis equestris* | Orchidaceae | Asparagales | CYC/TB1 |
| *PETCP06750* | PEQU_06750 | Orchidbase | *Phalaenopsis equestris* | Orchidaceae | Asparagales | CYC/TB1 |
| *PETCP11715* | PEQU_11715 | Orchidbase | *Phalaenopsis equestris* | Orchidaceae | Asparagales | CYC/TB1 |
| *PhavTCP1* | Phvul008G020400 | Phytozome | *Phaseolus vulgaris* | Fabaceae | Fabales | CYC/TB1 |
| *PhavTCP18* | Phvul009G200600 | Phytozome | *Phaseolus vulgaris* | Fabaceae | Fabales | CYC/TB1 |
| *PhavTCP18b* | Phvul003G177300 | Phytozome | *Phaseolus vulgaris* | Fabaceae | Fabales | CYC/TB1 |
| *PhavTCP1b* | Phvul007G060100 | Phytozome | *Phaseolus vulgaris* | Fabaceae | Fabales | CYC/TB1 |
| *PhavTCP1c* | Phvul004G072100 | Phytozome | *Phaseolus vulgaris* | Fabaceae | Fabales | CYC/TB1 |
| *PlorCYCL1* | HQ599283 | NCBI | *Platanus orientalis* | Platanaceae | Proteales | CYC/TB1 |
| *PlorCYCL2* | HQ599284 | NCBI | *Platanus orientalis* | Platanaceae | Proteales | CYC/TB1 |
| *PotrTCP1* | Potri.017G112000 | Phytozome | *Populus trichocarpa* | Salicaceae | Malpighiales | CYC/TB1 |
| *PotrTCP18* | Potri.015G050500 | Phytozome | *Populus trichocarpa* | Salicaceae | Malpighiales | CYC/TB1 |
| *PotrTCP18b* | Potri.012G059900 | Phytozome | *Populus trichocarpa* | Salicaceae | Malpighiales | CYC/TB1 |
| *PrhoCyL1* | DQ659321 | NCBI | *Papaver rhoeas* | Papaveraceae | Ranunculales | CYC/TB1 |
| *PrhoCyL2* | DQ659322 | NCBI | *Papaver rhoeas* | Papaveraceae | Ranunculales | CYC/TB1 |
| *PrupTCP1* | Ppa027156m.g | Phytozome | *Prunus persica* | Rosaceae | Rosales | CYC/TB1 |
| *PrupTCP12* | Ppa016240m.g | Phytozome | *Prunus persica* | Rosaceae | Rosales | CYC/TB1 |
| *RicoTCP1* | 30167.t000005 | Phytozome | *Ricinus communis* | Euphorbiaceae | Malpighiales | CYC/TB1 |
| *SbiTCP12* | Sb01g010690/XM_002466552 | Phytozome | *Sorghum bicolor* | Poaceae | Poales | CYC/TB1 |
| *SbiTCP12b* | Sb02g024450/XM_002460157 | Phytozome | *Sorghum bicolor* | Poaceae | Poales | CYC/TB1 |
| *ShCYCL1* | KP242028 | NCBI | *Saruma henryi* | Aristolochiaceae | Piperales | CYC/TB1 |
| *SlyTCP1* | Solyc04g006980 | Phytozome | *Solanum lycopersicum* | Solanaceae | Solanales | CYC/TB1 |
| *SlyTCP18* | Solyc06g069240 | Phytozome | *Solanum lycopersicum* | Solanaceae | Solanales | CYC/TB1 |
| *SlyTCP18b* | Solyc03g119770 | Phytozome | *Solanum lycopersicum* | Solanaceae | Solanales | CYC/TB1 |
| *SlyTCP1b* | Solyc05g009900 | Phytozome | *Solanum lycopersicum* | Solanaceae | Solanales | CYC/TB1 |
| *StuTCP12* | PGSC0003DMG400000823 | Phytozome | *Solanum tuberosum* | Solanaceae | Solanales | CYC/TB1 |
| *StuTCP18* | PGSC0003DMG400004054 | Phytozome | *Solanum tuberosum* | Solanaceae | Solanales | CYC/TB1 |
| *StuTCP18b* | PGSC0003DMG400005705 | Phytozome | *Solanum tuberosum* | Solanaceae | Solanales | CYC/TB1 |
| *ThecTCP1* | Thecc1EG019518t1 | Phytozome | *Theobroma cacao* | Malvaceae | Malvales | CYC/TB1 |
| *ThecTCP12* | Thecc1EG011874t1 | Phytozome | *Theobroma cacao* | Malvaceae | Malvales | CYC/TB1 |
| *ThecTCP18* | Thecc1EG01225t1 | Phytozome | *Theobroma cacao* | Malvaceae | Malvales | CYC/TB1 |
| *ThhaTCP1* | Thhalv10019499m.g. | Phytozome | *Thellungiella halophila* | Brassicaceae | Brassicales | CYC/TB1 |
| *ThhaTCP12* | Thhalv10018773m.g. | Phytozome | *Thellungiella halophila* | Brassicaceae | Brassicales | CYC/TB1 |
| *ThhaTCP18* | Thhalv10021948m.g. | Phytozome | *Thellungiella halophila* | Brassicaceae | Brassicales | CYC/TB1 |
| *TpTB1a* | JQ622132 | NCBI | *Tradescantia pallida* | Commelinaceae | Commelinales | CYC/TB1 |
| *TpTB1b* | JQ622133 | NCBI | *Tradescantia pallida* | Commelinaceae | Commelinales | CYC/TB1 |
| *VaTCP06749* | unigene179956_Va_fb | Orchidbase | *Vanilla shenzhenica* | Orchidaceae | Asparagales | CYC/TB1 |
| *ViviTCP1* | GSVIVG01011962001 | Phytozome | *Vitis vinifera* | Vitaceae | Vitales | CYC/TB1 |
| *ViviTCP18* | GSVIVG01008234001 | Phytozome | *Vitis vinifera* | Vitaceae | Vitales | CYC/TB1 |
| *ZemaTCP1/BAD1* | GRM2M26110242/JX122765 | Phytozome/NCBI | *Zea mays* | Poaceae | Poales | CYC/TB1 |
| *ZemaTCP12* | AC190734.2FG003/AF466646 | Phytozome/NCBI | *Zea mays* | Poaceae | Poales | CYC/TB1 |
| *ZemaTCP12b* | AC233950.1FG002/XM_008667055 | Phytozome/NCBI | *Zea mays* | Poaceae | Poales | CYC/TB1 |
| *ZemaTCP18b* | GRM2M26060319/BT063457 | Phytozome/NCBI | *Zea mays* | Poaceae | Poales | CYC/TB1 |
| *ZemaTCP18c* | GRM2M26055024/XM_008664286 | Phytozome/NCBI | *Zea mays* | Poaceae | Poales | CYC/TB1 |
| *ZemaTCP1b* | GRM2M26064628/NM_001196163 | Phytozome/NCBI | *Zea mays* | Poaceae | Poales | CYC/TB1 |
| *ZmTB1* | NM_001155479/U94494.1 | NCBI | *Zea mays* | Poaceae | Poales | CYC/TB1 |
| *AdTCP* | FGRF-2054791 | 1KP | *Asparagus densiflorus* | Asparagaceae | Asparagales | PCF |
| *AgaTCP-1* | PRFU-2130685 | 1KP | *Agapanthus africanus* | Amaryllidaceae | Asparagales | PCF |
| *AthTCP11* | At2g37000/NP_181237/NM_129256.2 | NCBI | *Arabidopsis thaliana* | Brassicaceae | Brassicales | PCF |
| *AthTCP14* | At3g47620/NP_190346/NM_114630.3 | NCBI | *Arabidopsis thaliana* | Brassicaceae | Brassicales | PCF |
| *AthTCP15* | At1g69690/NP_564973/NM_105637.2 | NCBI | *Arabidopsis thaliana* | Brassicaceae | Brassicales | PCF |
| *AthTCP16* | At3g45150/NP_190101/NM_114384.1 | NCBI | *Arabidopsis thaliana* | Brassicaceae | Brassicales | PCF |
| *AthTCP19* | At5g51910/NP_851173/NM_180842.1 | NCBI | *Arabidopsis thaliana* | Brassicaceae | Brassicales | PCF |
| *AthTCP20* | At3g27010/NP_189337/NM_113615.3 | NCBI | *Arabidopsis thaliana* | Brassicaceae | Brassicales | PCF |
| *AthTCP21* | At5g08330/NP_196450/NM_120916.2 | NCBI | *Arabidopsis thaliana* | Brassicaceae | Brassicales | PCF |
| *AthTCP22* | At1g72010/NP_177346/NM_105859.4 | NCBI | *Arabidopsis thaliana* | Brassicaceae | Brassicales | PCF |
| *AthTCP23* | At1g35560/NP_174789/NM_103253.2 | NCBI | *Arabidopsis thaliana* | Brassicaceae | Brassicales | PCF |
| *AthTCP6* | At5g41030/NP_198919/NM_123468.1 | NCBI | *Arabidopsis thaliana* | Brassicaceae | Brassicales | PCF |
| *AthTCP7* | At5g23280/NP_197719/NM_122234.2 | NCBI | *Arabidopsis thaliana* | Brassicaceae | Brassicales | PCF |
| *AthTCP8* | At1g58100/NP_1761077/NM_104592.4 | NCBI | *Arabidopsis thaliana* | Brassicaceae | Brassicales | PCF |
| *AthTCP9* | At2g45680/NP_182092/NM_130131.2 | NCBI | *Arabidopsis thaliana* | Brassicaceae | Brassicales | PCF |
| *AtrTCP4* | AmTr_v1.0_scaffold00069 | www.amborella.org | *Amborella trichopoda* | Amborellaceae | Amborellales | PCF |
| *AvTCP10* | JVBR-2044439 | 1KP | *Aloe vera* | Xanthorrhoeaceae | Asparagales | PCF |
| *AvTCP7* | JVBR-2014954 | 1KP | *Aloe vera* | Xanthorrhoeaceae | Asparagales | PCF |
| *AvTCP8* | JVBR-2009998 | 1KP | *Aloe vera* | Xanthorrhoeaceae | Asparagales | PCF |
| *BosTCP6* | EMJJ-2110546 | 1KP | *Borya sphaerocephala* | Boryaceae | Asparagales | PCF |
| *BosTCP8* | EMJJ-2018571 | 1KP | *Borya sphaerocephala* | Boryaceae | Asparagales | PCF |
| *BsTCP-1* | IXEM-2006200 | 1KP | *Brodiaea sierrae* | Asparagaceae | Asparagales | PCF |
| *BsTCP-3* | IXEM-2006201 | 1KP | *Brodiaea sierrae* | Asparagaceae | Asparagales | PCF |
| *BsTCP6* | IXEM-2067842 | 1KP | *Brodiaea sierrae* | Asparagaceae | Asparagales | PCF |
| *CoTCP-1* | KYNE-2004867 | 1KP | *Cyanella orchidofromis* | Tecophilaeaceae | Asparagales | PCF |
| *CtrPCF1* | c14669_g1_i1 | UdeA | *Cattleya trianae* | Orchidaceae | Asparagales | PCF |
| *CtrPCF10* | c24243_g4_i1 | UdeA | *Cattleya trianae* | Orchidaceae | Asparagales | PCF |
| *CtrPCF11* | c20858_g1_i1 | UdeA | *Cattleya trianae* | Orchidaceae | Asparagales | PCF |
| *CtrPCF2* | c10284_g1_i1 | UdeA | *Cattleya trianae* | Orchidaceae | Asparagales | PCF |
| *CtrPCF3* | c22906_g1_i1 | UdeA | *Cattleya trianae* | Orchidaceae | Asparagales | PCF |
| *CtrPCF4* | c10751_g2_i1 | UdeA | *Cattleya trianae* | Orchidaceae | Asparagales | PCF |
| *CtrPCF5* | c21320_g2_i2 | UdeA | *Cattleya trianae* | Orchidaceae | Asparagales | PCF |
| *CtrPCF6* | c23464_g2_i1 | UdeA | *Cattleya trianae* | Orchidaceae | Asparagales | PCF |
| *CtrPCF7* | c13613_g1_i1 | UdeA | *Cattleya trianae* | Orchidaceae | Asparagales | PCF |
| *CtrPCF8* | c8381_g1_i1 | UdeA | *Cattleya trianae* | Orchidaceae | Asparagales | PCF |
| *CtrPCF9* | c16850_g1_i1 | UdeA | *Cattleya trianae* | Orchidaceae | Asparagales | PCF |
| *CurTCP* | YJUG-2005390 | 1KP | *Curculigo sp.* | Hypoxidaceae | Asparagales | PCF |
| *CurTCP11* | YJUG-2137363 | 1KP | *Curculigo sp.* | Hypoxidaceae | Asparagales | PCF |
| *CurTCP9* | YJUG-62128354 | 1KP | *Curculigo sp.* | Hypoxidaceae | Asparagales | PCF |
| *CycTCP-1* | RDYY-2002847 | 1KP | *Cyanastrum cordifolium* | Tecophilaeaceae | Asparagales | PCF |
| *CycTCP-2* | RDYY-2002846 | 1KP | *Cyanastrum cordifolium* | Tecophilaeaceae | Asparagales | PCF |
| *DeTCP-1* | XZME-2015717 | 1KP | *Drakea elastica* | Orchidaceae | Asparagales | PCF |
| *DeTCP5* | XZME-2002957 | 1KP | *Drakea elastica* | Orchidaceae | Asparagales | PCF |
| *DpTCP-1* | UZXL-2029233 | 1KP | *Disporopsis pernyi* | Asparagaceae | Asparagales | PCF |
| *EpTCP-1* | EPTC000997 | Orchidstra | *Erycina pusilla* | Orchidaceae | Asparagales | PCF |
| *EpTCP-2* | EPTC012643 | Orchidstra | *Erycina pusilla* | Orchidaceae | Asparagales | PCF |
| *GpTCP-1* | VTUS-2009763 | 1KP | *Goodyera pubescens* | Orchidaceae | Asparagales | PCF |
| *GpTCP6* | VTUS-2049522 | 1KP | *Goodyera pubescens* | Orchidaceae | Asparagales | PCF |
| *HdTCP-2* | LELS-2014025 | 1KP | *Haemaria discolor* | Orchidaceae | Asparagales | PCF |
| *HdTCP5* | LELS-2087487 | 1KP | *Haemaria discolor* | Orchidaceae | Asparagales | PCF |
| *HemTCP-1* | JHOL-2013157 | 1KP | *Hemerocallis sp.* | Xanthorrhoeaceae | Asparagales | PCF |
| *HemTCP5* | JHUL-2006146 | 1KP | *Hemerocallis sp.* | Xanthorrhoeaceae | Asparagales | PCF |
| *HpTCP-4* | CMCY-2027527 | 1KP | *Hesperaloe parviflora* | Asparagaceae | Asparagales | PCF |
| *HydPCF1* | c25819_g1_i2 | UdeA | *Hypoxis decumbens* | Hypoxidaceae | Asparagales | PCF |
| *HydPCF10* | c27872_g2_i1 | UdeA | *Hypoxis decumbens* | Hypoxidaceae | Asparagales | PCF |
| *HydPCF2* | c25819_g1_i1 | UdeA | *Hypoxis decumbens* | Hypoxidaceae | Asparagales | PCF |
| *HydPCF3* | c29001_g5_i3 | UdeA | *Hypoxis decumbens* | Hypoxidaceae | Asparagales | PCF |
| *HydPCF4* | c29001_g5_i1 | UdeA | *Hypoxis decumbens* | Hypoxidaceae | Asparagales | PCF |
| *HydPCF5* | c29001_g5_i4 | UdeA | *Hypoxis decumbens* | Hypoxidaceae | Asparagales | PCF |
| *HydPCF6* | c29001_g5_i2 | UdeA | *Hypoxis decumbens* | Hypoxidaceae | Asparagales | PCF |
| *HydPCF7* | c27872_g4_i1 | UdeA | *Hypoxis decumbens* | Hypoxidaceae | Asparagales | PCF |
| *HydPCF8* | c27872_g3_i2 | UdeA | *Hypoxis decumbens* | Hypoxidaceae | Asparagales | PCF |
| *HydPCF9* | c27872_g3_i1 | UdeA | *Hypoxis decumbens* | Hypoxidaceae | Asparagales | PCF |
| *LlTCP-1* | MUMD-2018295 | 1KP | *Lomandra longifolia* | Asparagaceae | Asparagales | PCF |
| *McTCP-1* | XFFG-2022359 | 1KP | *Maianthemum canadense* | Asparagaceae | Asparagales | PCF |
| *McTCP-2* | XFFG-2007357 | 1KP | *Maianthemum canadense* | Asparagaceae | Asparagales | PCF |
| *McTCP-3* | XFJG-2007354 | 1KP | *Maianthemum canadense* | Asparagaceae | Asparagales | PCF |
| *MyTCP-2* | JSAG-2005139 | 1KP | *Masdevallia yungasensis* | Orchidaceae | Asparagales | PCF |
| *MyTCP-3* | JSAG-2011181 | 1KP | *Masdevallia yungasensis* | Orchidaceae | Asparagales | PCF |
| *NbTCP-3* | RQZP-2005587 | 1KP | *Nolina bigelovii* | Asparagaceae | Asparagales | PCF |
| *NbTCP-4* | RQZP-2005586 | 1KP | *Nolina bigelovii* | Asparagaceae | Asparagales | PCF |
| *NbTCP-5* | RQZP-2005588 | 1KP | *Nolina bigelovii* | Asparagaceae | Asparagales | PCF |
| *NbTCP8* | RQZP-2029896 | 1KP | *Nolina bigelovii* | Asparagaceae | Asparagales | PCF |
| *NvTCP-1* | TRRQ-2006855 | 1KP | *Narcissus viridiflorus* | Amaryllidaceae | Asparagales | PCF |
| *NvTCP-3* | XEUV-2021553 | 1KP | *Narcissus viridiflorus* | Amaryllidaceae | Asparagales | PCF |
| *OiTCP1* | comp16641 | De Paolo et. al 2015 | *Orchis italica* | Orchidaceae | Asparagales | PCF |
| *OiTCP2* | comp24776 | De Paolo et. al 2015 | *Orchis italica* | Orchidaceae | Asparagales | PCF |
| *OiTCP3* | comp13386 | De Paolo et. al 2015 | *Orchis italica* | Orchidaceae | Asparagales | PCF |
| *OiTCP4* | comp21123 | De Paolo et. al 2015 | *Orchis italica* | Orchidaceae | Asparagales | PCF |
| *OiTCP5* | comp12442 | De Paolo et. al 2015 | *Orchis italica* | Orchidaceae | Asparagales | PCF |
| *OiTCP6* | comp8378 | De Paolo et. al 2015 | *Orchis italica* | Orchidaceae | Asparagales | PCF |
| *OiTCP8* | comp21881 | De Paolo et. al 2015 | *Orchis italica* | Orchidaceae | Asparagales | PCF |
| *OiTCP9* | comp8964 | De Paolo et. al 2015 | *Orchis italica* | Orchidaceae | Asparagales | PCF |
| *OncTCP-1* | OGTC015462 | Orchidstra | *Onc. Gower ramsey* | Orchidaceae | Asparagales | PCF |
| *OncTCP-2* | OGTC041125 | Orchidstra | *Onc. Gower ramsey* | Orchidaceae | Asparagales | PCF |
| *OncTCP-4* | OGTC021642 | Orchidstra | *Onc. Gower ramsey* | Orchidaceae | Asparagales | PCF |
| *OncTCP-5* | OGTC015461 | Orchidstra | *Onc. Gower ramsey* | Orchidaceae | Asparagales | PCF |
| *OsPCF1* | LOC_Os04g11830 | Phytozome | *Oryza sativa* | Poaceae | Poales | PCF |
| *OsPCF2* | LOC_Os08g43160 | Phytozome | *Oryza sativa* | Poaceae | Poales | PCF |
| *OsPCF3* | LOC_Os11g07460 | Phytozome | *Oryza sativa* | Poaceae | Poales | PCF |
| *OsTCP17* | LOC_Os04g44440 | Phytozome | *Oryza sativa* | Poaceae | Poales | PCF |
| *OsTCP19* | LOC_Os06g12230 | Phytozome | *Oryza sativa* | Poaceae | Poales | PCF |
| *OsTCP25* | LOC_Os09g34950 | Phytozome | *Oryza sativa* | Poaceae | Poales | PCF |
| *OsTCP28* | LOC_Os12g07480 | Phytozome | *Oryza sativa* | Poaceae | Poales | PCF |
| *OsTCP6* | LOC_Os01g69980 | Phytozome | *Oryza sativa* | Poaceae | Poales | PCF |
| *OsTCP7* | LOC_Os02g42380 | Phytozome | *Oryza sativa* | Poaceae | Poales | PCF |
| *OsTCP9* | LOC_Os02g51280 | Phytozome | *Oryza sativa* | Poaceae | Poales | PCF |
| *PaTCP-1* | PATC157577 | Orchidstra | *Phalaenopsis aphrodite* | Orchidaceae | Asparagales | PCF |
| *PaTCP-2* | PATC125299 | Orchidstra | *Phalaenopsis aphrodite* | Orchidaceae | Asparagales | PCF |
| *PaTCP-4b* | PATC124397 | Orchidstra | *Phalaenopsis aphrodite* | Orchidaceae | Asparagales | PCF |
| *PcTCP1* | DMIN-2087680 | 1KP | *Phycella aff. Cyrtanthoides* | Amaryllidaceae | Asparagales | PCF |
| *PemTCP-1* | TCYS-2093796 | 1KP | *Peliosanthese minor* | Asparagaceae | Asparagales | PCF |
| *PETCP03822* | PEQU_03822 | Orchidbase | *Phalaenopsis equestris* | Orchidaceae | Asparagales | PCF |
| *PETCP09751* | PEQU_09751 | Orchidbase | *Phalaenopsis equestris* | Orchidaceae | Asparagales | PCF |
| *PeTCP-1* | PETC016471 | Orchidstra | *Phalaenopsis equestris* | Orchidaceae | Asparagales | PCF |
| *PETCP15634* | PEQU_15634 | Orchidbase | *Phalaenopsis equestris* | Orchidaceae | Asparagales | PCF |
| *PETCP19398* | PEQU_19398 | Orchidbase | *Phalaenopsis equestris* | Orchidaceae | Asparagales | PCF |
| *PeTCP-2* | PETC020806 | Orchidstra | *Phalaenopsis equestris* | Orchidaceae | Asparagales | PCF |
| *PETCP21260* | PEQU_21260 | Orchidbase | *Phalaenopsis equestris* | Orchidaceae | Asparagales | PCF |
| *PETCP25177* | PEQU_25177 | Orchidbase | *Phalaenopsis equestris* | Orchidaceae | Asparagales | PCF |
| *PETCP28429* | PEQU_28429 | Orchidbase | *Phalaenopsis equestris* | Orchidaceae | Asparagales | PCF |
| *PETCP39718* | PEQU_39718 | Orchidbase | *Phalaenopsis equestris* | Orchidaceae | Asparagales | PCF |
| *PETCP40981* | PEQU_40981 | Orchidbase | *Phalaenopsis equestris* | Orchidaceae | Asparagales | PCF |
| *PeTCPb* | singletons 17327/54932 | Orchidbase | *Phalaenopsis equestris* | Orchidaceae | Asparagales | PCF |
| *PhaTCP-4* | HQ439606.1 | NCBI | *Phalaenopsis hybrid cult.* | Orchidaceae | Asparagales | PCF |
| *PmTCP-1* | PMTC018572 | Orchidstra | *Phalaenopsis modesta* | Orchidaceae | Asparagales | PCF |
| *PmTCP-2* | PMTC004124 | Orchidstra | *Phalaenopsis modesta* | Orchidaceae | Asparagales | PCF |
| *RusTCP4* | LSJW-2088537 | 1KP | *Ruscus sp.* | Asparagaceae | Asparagales | PCF |
| *StTCP-2* | MVRF-2005143 | 1KP | *Sansevieria trifasciata* | Asparagaceae | Asparagales | PCF |
| *StTCP9* | MVRF-2005142 | 1KP | *Sansevieria trifasciata* | Asparagaceae | Asparagales | PCF |
| *TmTCP-1* | ZKPF-2002490 | 1KP | *Traubia modesta* | Amaryllidaceae | Asparagales | PCF |
| *TmTCP-2* | ZKPF-2007378 | 1KP | *Traubia modesta* | Amaryllidaceae | Asparagales | PCF |
| *VpTCP5* | THDM-2011391 | 1KP | *Vanilla planifolia* | Orchidaceae | Asparagales | PCF |
| *XcTCP11* | SART-2044918 | 1KP | *Xeronema callistemon* | Xeronemataceae | Asparagales | PCF |
| *XcTCP-2* | SART-2007406 | 1KP | *Xeronema callistemon* | Xeronemataceae | Asparagales | PCF |
| *XcTCP-3* | SART-2007405 | 1KP | *Xeronema callistemon* | Xeronemataceae | Asparagales | PCF |
| *XcTCP-4* | SART-2007408 | 1KP | *Xeronema callistemon* | Xeronemataceae | Asparagales | PCF |
| *YfTCP-3* | ICNN-2001528 | 1KP | *Yucca filamentosa* | Asparagaceae | Asparagales | PCF |
| *ZtTCP-1* | DPFW-2005965 | 1KP | *Zephyranthes treatiae* | Amaryllidaceae | Asparagales | PCF |
| *SlyTCP4* | Solyc03g006800.1 | Phytozome | *Solanum lycopersicum* | Solanaceae | Solanales | PCF |
| *StuTCP4* | PGSC0003DMT400062327 | Phytozome | *Solanum tuberosum* | Solanaceae | Solanales | PCF |
| *SlyTCP15* | Solyc06g070900.2.1 | Phytozome | *Solanum lycopersicum* | Solanaceae | Solanales | PCF |
| *StuTCP15* | PGSC0003DMT400083148 | Phytozome | *Solanum tuberosum* | Solanaceae | Solanales | PCF |
| *SlyTCP11* | Solyc01g008230.2.1 | Phytozome | *Solanum lycopersicum* | Solanaceae | Solanales | PCF |
| *StuTCP11* | PGSC0003DMT400042166 | Phytozome | *Solanum tuberosum* | Solanaceae | Solanales | PCF |
| *SlyTCP23* | Solyc11g020670.1.1 | Phytozome | *Solanum lycopersicum* | Solanaceae | Solanales | PCF |
| *StuTCP23* | PGSC0003DMT400023981 | Phytozome | *Solanum tuberosum* | Solanaceae | Solanales | PCF |
| *SlyTCP6* | Solyc05g007420.1.1 | Phytozome | *Solanum lycopersicum* | Solanaceae | Solanales | PCF |
| *StuTCP6* | PGSC0003DMT400008728 | Phytozome | *Solanum tuberosum* | Solanaceae | Solanales | PCF |
| *SlyTCP20* | Solyc02g068200.1.1 | Phytozome | *Solanum lycopersicum* | Solanaceae | Solanales | PCF |
| *StuTCP20* | PGSC0003DMT400090661 | Phytozome | *Solanum tuberosum* | Solanaceae | Solanales | PCF |
| *SlyTCP19* | Solyc08g080150.1.1 | Phytozome | *Solanum lycopersicum* | Solanaceae | Solanales | PCF |
| *StuTCP19* | PGSC0003DMT400058534 | Phytozome | *Solanum tuberosum* | Solanaceae | Solanales | PCF |
| *SlyTCP8* | Solyc06g065190.1.1 | Phytozome | *Solanum lycopersicum* | Solanaceae | Solanales | PCF |
| *StuTCP8* | PGSC0003DMT400067150 | Phytozome | *Solanum tuberosum* | Solanaceae | Solanales | PCF |
| *SlyTCP14* | Solyc03g116320.2.1 | Phytozome | *Solanum lycopersicum* | Solanaceae | Solanales | PCF |
| *StuTCP14* | PGSC0003DMT400001613 | Phytozome | *Solanum tuberosum* | Solanaceae | Solanales | PCF |

**Supplementary table 2.** Primers used for TCP-like gene expression analyses.

|  | **Fwd** | **Tm**  **Fwd** | **Rev** | **Tm**  **Rev** | **Amplicon**  **size (nd)** |
| --- | --- | --- | --- | --- | --- |
| **HydTB1** | GGGCCTCGGGACCGGCGGA | 70 | CTCGCCCGAGCCTTGTCTCTAG | 72 | 299 |
| **HydCIN1** | CGTCCTCGAGGATCTACCG | 62 | GAAGGAGCTCTGAGAGTTGAG | 64 | 481 |
| **HydCIN2** | CGGAACGATGTCAACATCAAG | 62 | GACGGTTGTATCGCGTGGTTC | 66 | 552 |
| **HydCIN3** | CCTCCTCCTCCTCCGCTTG | 64 | GTGGGTGGGGGGCGGGGTG | 66 | 225 |
| **HydCIN4** | CACATTGTCCGGTCGACCGA | 64 | CGAGAATCCGATGGCCGGAAT | 66 | 629 |
| **HydPCF1** | CGACGAGGCGGGAAAGAAG | 62 | CAGCTGAAGCTCCTGCTTCAC | 66 | 426 |
| **HydPCF2** | CGATTCCGGTGCACCGCCT | 64 | CATCAGCTGAAGCTCCTGCTT | 64 | 794 |
| **HydPCF3** | ATGGATTCCCACAGCGAGGAA | 64 | GGAGCCGGTGGCGGCGATGAT | 72 | 268 |
| **HydPCF4** | ATGGATTCCCACAGCGAGGAA | 64 | GGAGCCGGTGGCGGCGATGAT | 72 | 265 |
| **HydPCF5** | CAACTCCAAGTTCCATCAGGC | 64 | GGAGCCGGTGGCGGCGATGAT | 72 | 272 |
| **HydPCF6** | CAACTCCAAGTTCCATCAGGC | 64 | GGAGCCGGTGGCGGCGATGAT | 72 | 269 |
| **HydPCF7** | ACTAACGGCCTAGCCGACCA | 64 | CTAAATCAGCGGTTCTCCTC | 60 | 716 |
| **HydPCF8** | ACTAACGGCCTAGCCGACCA | 64 | CATATCAGCGAGGATCCTCCT | 64 | 705 |
| **HydPCF9** | ATGGCGACGGCTGACGGAG | 64 | CATATCAGCGAGGATCCTCCT | 64 | 721 |
| **HydPCF10** | CAAACCCGCCCCGGCTCCTC | 70 | GTTTAGATCCCGTCGGCGA | 60 | 580 |
| **CtrTB1** | CAATGGCTGCTCAACAAATCG | 62 | CAAACACTGGTGGTATTATTGG | 62 | 558 |
| **CtrCIN1** | GGAGAGATCGTCGAGGTTCAG | 66 | GCCTGCTGCATTTGGTGATC | 62 | 738 |
| **CtrCIN2** | GAGATCGTAGAAGTACAAGGC | 62 | CTGTATTCGTGCAGGTATATG | 60 | 829 |
| **CtrCIN3** | CTGAACACCAGATCTCGCCG | 64 | CAGTCTTCAGAACCAGTTAACAG | 66 | 768 |
| **CtrCIN4** | CTCCGAACTGTCCTTCACCAG | 66 | CAGGCATGTCCACCACATAGA | 64 | 874 |
| **CtrCIN5** | CGCAACATGGTCTTATCTCAA | 60 | CTGGCAAGACCTGGTGCGATG | 68 | 770 |
| **CtrCIN6** | GAACCGAAGAACCATCGCTTC | 64 | CTTGACCGCTTGTGCTCATCG | 66 | 597 |
| **CtrPCF1** | CATCGACAGCGATGACTACG | 62 | CTTCATGCCCACGTTTCTGAT | 62 | 755 |
| **CtrPCF2** | CTCCAGAGAAGGAGTTGGAAG | 64 | CTCTGCCGTCTCCCTCATCAA | 66 | 642 |
| **CtrPCF3** | GTCCAAGTTGCAGCATCTTCC | 64 | CATATTAGGATGAACAATTAAG | 56 | 862 |
| **CtrPCF4** | ATGCCGGCTCTCTGCGCCGCC | 74 | GTCCCATCTGCAGATAAAACT | 60 | 521 |
| **CtrPCF5** | CAGAGACCTCCAAGTGCTCGT | 66 | GATGATGCTGTAACTGTTCAC | 60 | 699 |
| **CtrPCF6** | GTGAACGGTGGGAACGGTAA | 62 | GTAAGAAAATCAGCGAGGCTC | 62 | 734 |
| **CtrPCF7** | GGTAATGGAAAGAAGAGCGAG | 62 | CAAGCAGGTTAAGATGCGCAT | 62 | 563 |
| **CtrPCF8** | CTCTTTCTCTCATGCCATCT | 58 | CCTAAAAGAAGCAATCTGCTA | 58 | 605 |
| **CtrPCF9** | GACTCATCCCTCGCCATTTCA | 64 | CTGAGAATTGCTATGCCCATC | 62 | 901 |
| **CtrPCF10** | ACGTTGGAGGAGGAGGTCCT | 64 | CTAAGAGTCGCTGGTGCTCAT | 64 | 1054 |
| **CtrPCF11** | CCACATCAGTAACAGGACTCA | 62 | CAACCGGTCTATACGAGTTAA | 60 | 682 |
